# Lifestyle interventions and 24-hour movement behaviors in preschool children: a systematic review and meta-analysis

**DOI:** 10.3389/fpubh.2026.1846736

**Published:** 2026-06-17

**Authors:** Yu Wu, Marcin Białas, Yintao Niu, Sujie Mao, Zbigniew Ossowski, Qian Zhou, Lvwei Yu, Zhiyi Wang, Ansu Zhong, Guoping Qian

**Affiliations:** 1 Department of Exercise and Health, Guiyang Preschool Education College, Guiyang, Guizhou, China; 2 Department of Physical Culture, Gdansk University of Physical Education and Sport, Gdansk, Poland; 3 Department of Physical Education, Chizhou University, Chizhou, China; 4 Graduate Development Office, Harbin Sport Institute, Harbin, China; 5 College of Physical Education and Health, Guizhou Minzu University, Guiyang, China; 6 Zunyi No. 2 Experimental Kindergarten, Zunyi, China; 7 Zunyi No. 1 Experimental Kindergarten, Zunyi, China

**Keywords:** 24-h movement behaviors, lifestyle interventions, physical activity, preschool children, screen time, sedentary behavior, sleep duration

## Abstract

**Background:**

Unhealthy 24-h movement behaviors, including insufficient physical activity, excessive sedentary behavior, excessive screen time, and inadequate sleep, are common among preschool children. However, evidence for the effectiveness of lifestyle interventions across these behavioral domains remains limited and inconsistent. This study aimed to evaluate the effects of lifestyle interventions on 24-h movement behaviors in preschool children.

**Methods:**

PubMed, SPORTDiscus, Scopus, Web of Science Core Collection, and CENTRAL were searched from inception to December 31, 2025. Randomized controlled trials involving children aged 2–6 years that evaluated structured lifestyle interventions targeting movement behavior domains were included. Random-effects meta-analyses were performed to estimate pooled mean differences (MDs) and 95% confidence intervals (CIs). Risk of bias was assessed using the Cochrane Risk of Bias 2 (RoB 2) tool, and certainty of evidence was assessed using the Grading of Recommendations, Assessment, Development, and Evaluation (GRADE) approach.

**Results:**

Forty-three trials from 16 countries were included, comprising 13,659 preschool children. Lifestyle interventions were associated with increases in moderate-to-vigorous physical activity (MD 5.77 min/day, 95% CI 2.27 to 9.28; I^2^ = 91%) and sleep duration (MD 0.18 h/day, 95% CI 0.01 to 0.35; I^2^ = 68%), and with reductions in sedentary behavior (MD − 7.62 min/day, 95% CI − 15.08 to −0.17; I^2^ = 63%) and screen time (MD − 0.33 h/day, 95% CI − 0.53 to −0.13; I^2^ = 95%). Effects on total physical activity and other intensity-specific physical activity outcomes remained uncertain. The overall risk of bias was rated as low in 2 trials, as having some concerns in 32 trials, and as high in 9 trials. The certainty of evidence was low for the primary outcomes, and small-study effects could not be ruled out for screen time.

**Conclusion:**

Lifestyle interventions were associated with modest improvements in several 24-h movement behaviors in preschool children. However, heterogeneity was substantial across outcomes, and the certainty of evidence was low. These findings should therefore be interpreted as favorable but uncertain directions of effect rather than precise estimates of intervention efficacy. Future trials should adopt more standardized measurement and reporting methods, include longer follow-up, and examine time reallocation within the 24-h movement composition.

**Systematic review registration:**

https://www.crd.york.ac.uk/PROSPERO/view/CRD420261332463, Identifier CRD420261332463.

## 1 Introduction

The preschool years represent a critical developmental window during which lifestyle and health-related behaviors are established rapidly and may persist into school age and adulthood (1, 2). Unhealthy movement behaviors are common in early childhood, including insufficient physical activity (PA), excessive sedentary behavior (SB), excessive recreational screen time (ST), and insufficient sleep duration (3, 4). These behaviors are associated with childhood obesity and a range of adverse health outcomes and may shape health trajectories later in life (5, 6). Within the “24-hour movement behavior” paradigm, the World Health Organization (WHO) and guidelines from several countries emphasize that PA, SB, and sleep should be considered together within a finite 24-h time budget (7). Accordingly, current recommendations for preschool children call for adequate daily PA, limited ST, and sufficient sleep (7–12). However, adherence to the integrated 24-h movement guidelines remains low worldwide. Tapia-Serrano et al. reported low international prevalence of meeting the combined 24-h movement guidelines across pediatric populations, underscoring the public health relevance of promoting healthy movement behaviors from early life onward (13). Consistent with this broader pattern, pooled data from 33 countries indicate that only about 13% of preschool children meet the combined recommendations for PA, SB, and sleep simultaneously (14, 15). Even for a single target, screen exposure, a systematic review and meta-analysis estimated that only about 35.6% of preschool children meet the recommendation of ≤1 h/day of ST (16).

Longitudinal studies suggest that moderate-to-vigorous physical activity (MVPA) declines and SB increases after school entry, underscoring the importance of intervention during the preschool years, when behavioral plasticity may be greater (17). Within a fixed 24-h time budget, increasing PA, reducing SB and ST, and promoting healthy sleep may confer broad health benefits in preschool children. Such changes may improve not only weight status and cardiometabolic health but also mental health, health-related quality of life, and developmental outcomes such as motor competence (18–21). Given the likelihood that early-life behaviors persist over time, the preschool period represents an important window for preventive intervention (22).

Lifestyle interventions are commonly defined as structured strategies aiming to modify unhealthy daily behavior patterns through components such as education, family support, and environmental modification (23). However, it remains unclear whether lifestyle interventions produce meaningful improvements across 24-h movement behaviors in preschool children. Previous trials and reviews have often focused on adiposity outcomes or a single behavioral domain, resulting in fragmented evidence, heterogeneous outcome definitions, and inconsistent reporting, which limit quantitative comparability across studies (24–26). More recently, a systematic review and meta-analysis of school-based interventions across preschoolers, children, and adolescents reported small favorable effects on sedentary behavior-related outcomes and sleep duration, whereas effects on physical activity outcomes were less consistent (27). However, that review focused specifically on school-based delivery across a broader age range, leaving a need for a synthesis focused on preschool children and lifestyle interventions delivered across multiple contexts, including preschool, family, and community settings. Because time within a day is finite, assessing PA, SB, and sleep in isolation may obscure their interdependence and the compensatory reallocation of time within the 24-h day. This interdependence is central to the 24-h movement behavior paradigm and has direct implications for intervention design and the prioritization of behavioral targets (28). Intervention effects may also vary according to intervention components and implementation context, such as caregiver involvement, setting, delivery mode, and intervention duration, thereby contributing to heterogeneity across trials (29, 30). In addition, inconsistent reporting and the limited availability of harmonized effect estimates can hinder the quantitative synthesis needed to inform the selection of intervention combinations and the prioritization of lifestyle targets (31, 32).

Therefore, this study evaluated the effects of lifestyle interventions, compared with usual care or control conditions, on 24-h movement behaviors in preschool children. The primary outcomes were MVPA, SB, ST, and sleep duration. The aim was to quantify pooled effects across behavioral domains within a 24-h time-budget framework and to inform the design and implementation of integrated interventions for preschool children.

## 2 Methods

This review was reported in accordance with PRISMA 2020 (Supplementary Tables 1–3) and conducted in accordance with the Cochrane Handbook for Systematic Reviews of Interventions. The review was registered in PROSPERO (CRD420261332463) (33–35).

### 2.1 Eligibility criteria

Study eligibility was prespecified using the PICOS framework (Population, Intervention, Comparator, Outcomes, and Study Design) (36).

#### 2.1.1 Population

We included preschool children aged 2–6 years, with no restrictions on sex, ethnicity, geographic region, or socioeconomic background (26, 37). Studies with broader age ranges were eligible if (i) the mean age fell within 2–6 years or (ii) extractable outcome data were available for a subgroup of children aged 2–6 years.

#### 2.1.2 Interventions

Eligible interventions were structured programs targeting lifestyle modification, consistent with WHO guidance emphasizing health improvement through changes in daily behavioral patterns (38, 39). To align with the 24-h movement behavior paradigm, interventions were required to target at least one relevant domain (PA, SB, ST, or sleep duration) and demonstrate an explicit intent to improve daily movement behavior patterns rather than merely provide health information. Interventions were required to provide sufficient detail on core components, delivery mode, setting (e.g., preschool/kindergarten, home, or community), dose (frequency and session duration, where applicable), and overall duration to permit classification and replication. We excluded interventions limited to general health information or single-session education without ongoing implementation support that could plausibly influence daily routines, such as repeated sessions, home practice materials, follow-up contacts, or environmental facilitation.

#### 2.1.3 Comparators

Comparators included usual care or standard practice, minimal intervention, wait-list control, or attention control, provided that they did not include an active, structured lifestyle program targeting the same behavioral domains as the intervention.

#### 2.1.4 Outcomes

The primary outcomes were MVPA, ST, SB, and sleep duration. Secondary outcomes included total physical activity (TPA), light physical activity (LPA), moderate physical activity (MPA), and vigorous physical activity (VPA). Outcomes could be assessed using device-based measures (e.g., accelerometers or actigraphy) or parent-reported instruments (e.g., questionnaires or diaries).

#### 2.1.5 Study design

We included individually randomized controlled trials (RCTs) and cluster-randomized controlled trials (CRCTs).

### 2.2 Data sources and search strategy

We searched PubMed, SPORTDiscus, Scopus, Web of Science Core Collection, and CENTRAL from inception to December 31, 2025. Search strategies were informed by previous reviews and refined for the current research question (26, 38, 39). The search strategy combined controlled vocabulary (e.g., MeSH terms and subject headings) with free-text terms related to “preschool children,” “lifestyle interventions,” “physical activity,” “sedentary behavior,” “screen time,” and “sleep duration.” Full search strategies for all databases are provided in Supplementary Table 4. We also screened the reference lists of included studies and relevant reviews and conducted forward citation tracking to identify additional studies.

### 2.3 Study selection

Records were imported into Rayyan for deduplication and screening management (40). Duplicates were removed using Rayyan’s automated detection tools, supplemented by manual verification. Two reviewers (YW and GPQ) independently screened titles and abstracts and assessed full texts against the eligibility criteria. Discrepancies were resolved through discussion, and unresolved cases were adjudicated by a third reviewer (MB).

### 2.4 Data extraction

A standardized data-extraction form was developed and pilot-tested. We extracted study identifiers, study design, participant characteristics, intervention and comparator characteristics (including core components, dose, setting, and delivery mode), outcome definitions, measurement methods, and outcome data for prespecified behaviors (primary outcomes: MVPA, SB, ST, and sleep duration; secondary outcomes: TPA, LPA, MPA, and VPA). When data were missing or unclear, we contacted the study authors. If data were unavailable, we applied prespecified data conversion methods and documented all assumptions. Two reviewers (YW and GPQ) independently extracted the data. Discrepancies were resolved through discussion, with arbitration by a third reviewer (MB) for key fields when necessary.

### 2.5 Data synthesis

For outcomes reported at both baseline and post-intervention, change scores (post-intervention minus baseline) were preferentially used to estimate intervention effects. The primary analyses used outcomes measured immediately after the intervention. When multiple post-intervention time points were reported, the time point closest to intervention completion was selected, and longer-term follow-up outcomes were synthesized separately when data permitted. If the SD of the change score was not reported, it was derived using Cochrane methods based on baseline and post-intervention SDs, assuming a within-person correlation coefficient of r = 0.5 between time points (34, 41).


$${\mathrm{SD}}_{\text{change}}=\sqrt{{\mathrm{SD}}_{\text{baseline}}^{2}+{\mathrm{SD}}_{\text{post}}^{2}-2\times r\times {\mathrm{SD}}_{\text{baseline}}\times {\mathrm{SD}}_{\text{post}}}$$

When variability was reported as a standard error (SE) or 95% confidence interval (CI), it was converted to an SD using standard formulas before pooling. When weekday and weekend means were reported separately, we calculated an overall daily mean using a prespecified weighted average.


$$\begin{array}{l} \text{Average daily outcome}\\ =\frac{\left(\text{Weekday outcome}\times 5)+(\text{Weekend outcome}\times 2\right)}{7} \end{array}$$

For CRCTs, we preferentially extracted effect estimates that accounted for clustering. When cluster-adjusted estimates were unavailable, clustering was adjusted using the design effect if the intraclass correlation coefficient (ICC) and average cluster size were available or could be reasonably derived; otherwise, unadjusted data were used, and potential unit-of-analysis errors (i.e., overly narrow CIs) were noted, with plausible ICC values explored in sensitivity analyses where feasible. For continuous outcomes, mean differences (MDs) were used when the same scale was applied, and Hedges’ g [i.e., standardized mean differences (SMDs) with a small-sample correction] was used when scales differed; pooled effects were reported with 95% CIs. Random-effects meta-analysis was used to account for between-study heterogeneity in intervention components, implementation contexts, and measurement approaches. Statistical heterogeneity was assessed using I^2^ and Cochran’s Q and interpreted alongside clinical and methodological diversity. When quantitative pooling was inappropriate or heterogeneity could not be plausibly explained, findings were summarized narratively. To assess the robustness of the findings, leave-one-out sensitivity analyses were conducted. Where data permitted, prespecified subgroup analyses were conducted according to measurement method. When at least 10 studies were available for an outcome, small-study effects were assessed using funnel plots and Egger’s and Begg’s tests. When fewer than 10 studies were available, formal tests were not performed, and this limitation was noted. Statistical analyses were conducted using Stata/MP version 14.1 (StataCorp, College Station, TX, USA) and Review Manager version 5.3 (Cochrane Collaboration), and results were interpreted primarily on the basis of effect sizes and 95% CIs.

### 2.6 Risk of bias

Risk of bias was assessed using the Cochrane Risk of Bias 2 (RoB 2) tool (42). Two reviewers (YW and GPQ) independently assessed risk of bias and documented domain-level justifications. Disagreements were resolved through discussion, and unresolved cases were adjudicated by a third reviewer (MB).

### 2.7 Certainty of the evidence

The GRADE framework was used to rate the certainty of the evidence for each outcome (43). Two reviewers independently rated the certainty of the evidence, with discrepancies resolved through discussion and adjudication when required.

### 2.8 Ethical considerations

This review synthesized published data and did not involve new participant recruitment or identifiable individual-level data; therefore, ethics approval and informed consent were not required.

## 3 Results

### 3.1 Study selection

**Figure 1:**
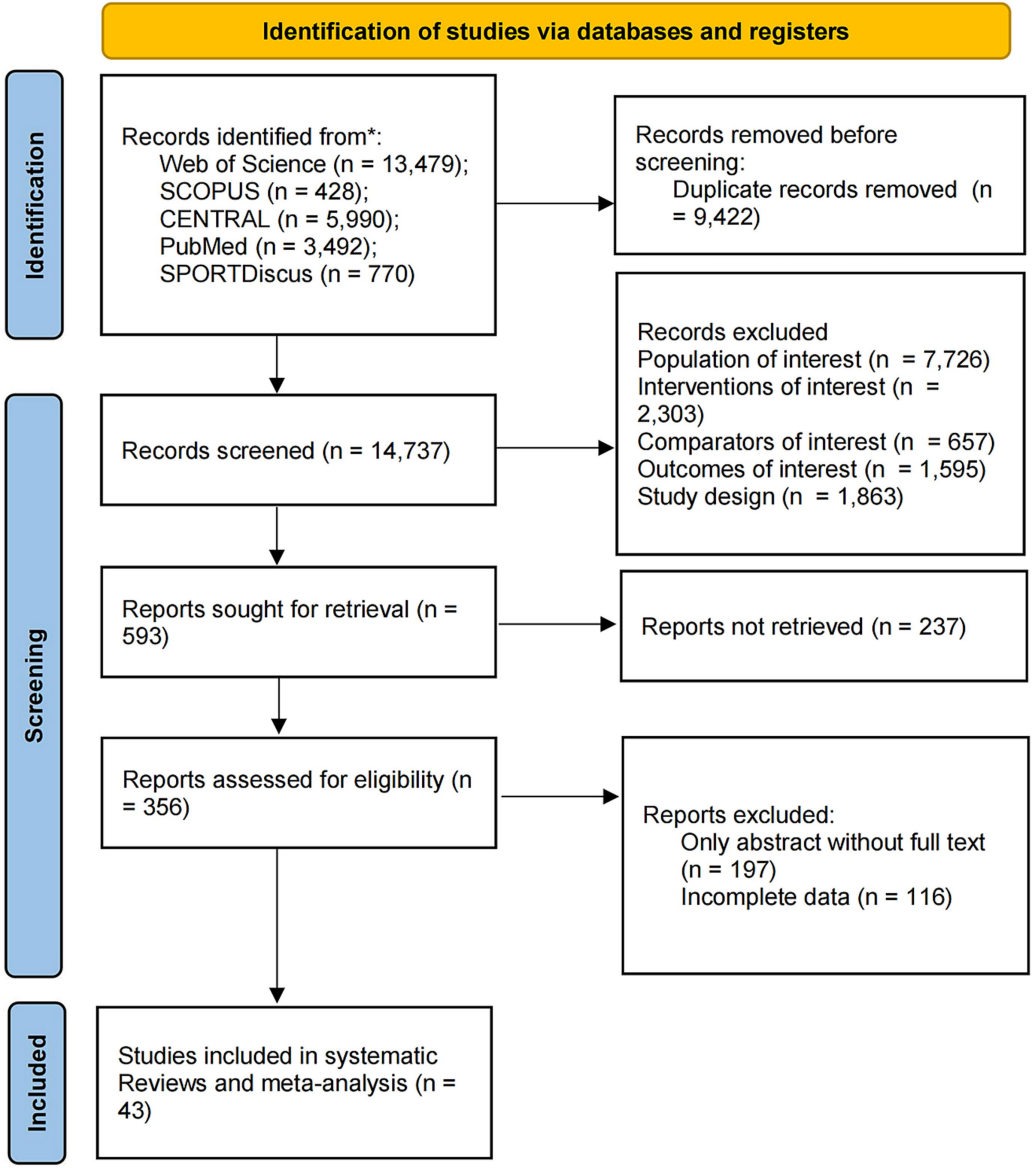
PRISMA flow diagram for selection of studies. PRISMA, Preferred Reporting Items for Systematic Reviews and Meta-Analyses.

The database searches identified 24,159 records (PubMed *n* = 3,492; Scopus *n* = 428; Web of Science *n* = 13,479; CENTRAL *n* = 5,990; SPORTDiscus *n* = 770). After deduplication and title and abstract screening, potentially relevant reports underwent full-text assessment, and 43 trials were ultimately included in the review. The numbers of records screened and excluded at each stage, including reasons for full-text exclusion, are shown in the PRISMA flow diagram (Figure 1).

### 3.2 Study characteristics

The characteristics of the included studies are summarized in Supplementary Table 5. Forty-three trials conducted across 16 countries were included, with sample sizes ranging from 22 to 1,589 preschool children (total *N* = 13,659). Most studies reported sex distribution by group, and the overall proportion of girls was approximately balanced. Interventions were predominantly multicomponent lifestyle programs, most commonly combining PA promotion with components targeting SB and ST; some also included sleep-related components. Comparator conditions most commonly involved usual care or standard practice. For outcome assessment, 25 studies used device-based measures (e.g., accelerometry or actigraphy), whereas 18 relied primarily on parent-reported instruments (e.g., questionnaires or diaries).

### 3.3 Meta-analyses

#### 3.3.1 Primary outcomes

##### 3.3.1.1 Moderate-to-vigorous physical activity

**Figure 2:**
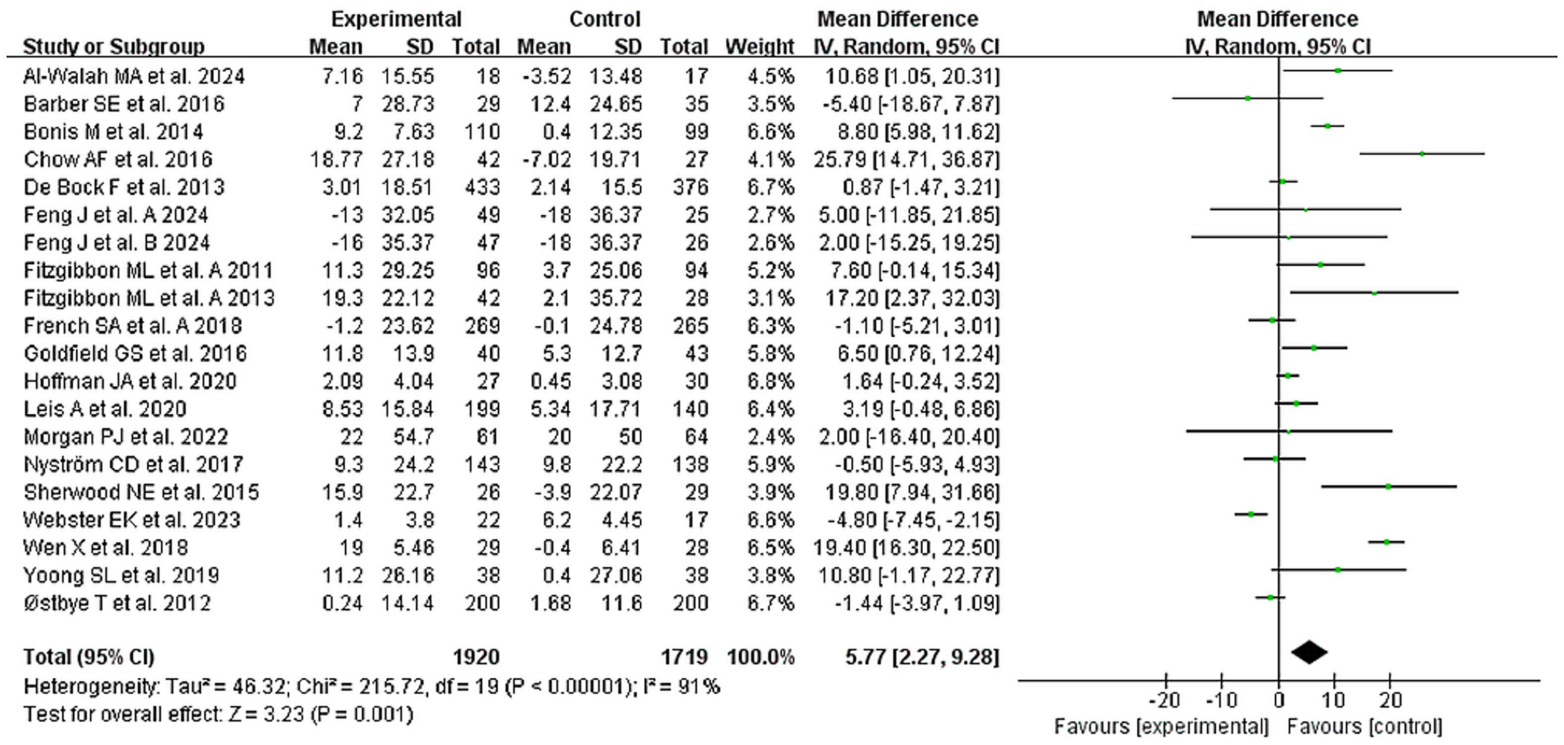
Forest plot of the effects of lifestyle interventions on MVPA. CI, confidence interval; Chi^2^, chi-square; df, degrees of freedom; I^2^, I-squared; IV, inverse variance; MVPA, moderate-to-vigorous physical activity; *P*, *p* value; SD, standard deviation; Tau^2^, between-study variance.

Twenty trials (*n* = 3,639; intervention, *n* = 1,920; control, *n* = 1,719) contributed to the MVPA meta-analysis. Compared with controls, lifestyle interventions were associated with a modest increase in MVPA (MD 5.77 min/day, 95% CI 2.27 to 9.28; I^2^ = 91%) (Figure 2).

##### 3.3.1.2 Sedentary behavior

**Figure 3:**
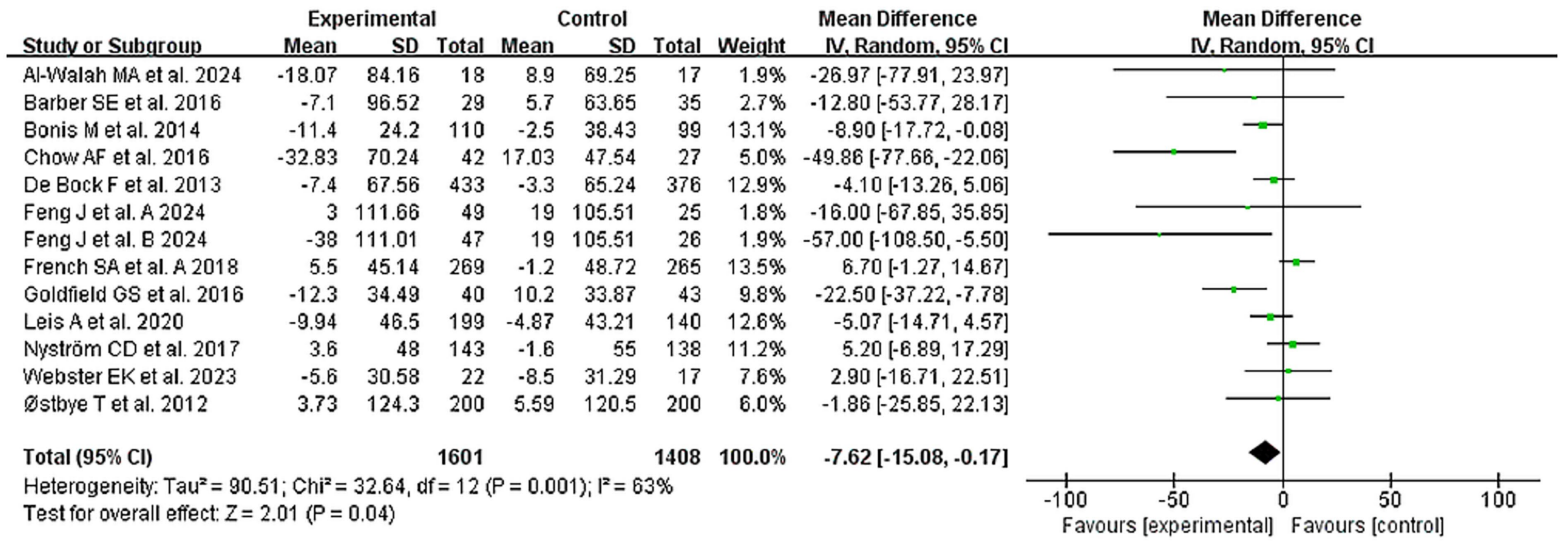
Forest plot of the effects of lifestyle interventions on SB. CI, confidence interval; Chi^2^, chi-square; df, degrees of freedom; I^2^, I-squared; IV, inverse variance; P, *p* value; SD, standard deviation; SB, sedentary behavior; Tau^2^, between-study variance.

Thirteen trials (*n* = 3,009; intervention, *n* = 1,601; control, *n* = 1,408) reported SB. Compared with controls, lifestyle interventions were associated with a small reduction in SB (MD − 7.62 min/day, 95% CI − 15.08 to −0.17; I^2^ = 63%) (Figure 3).

##### 3.3.1.3 Sleep duration

**Figure 4:**
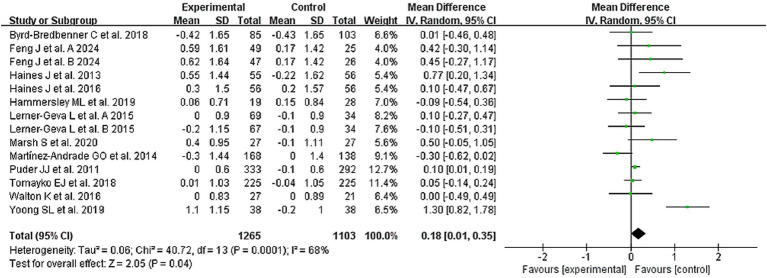
Forest plot of the effects of lifestyle interventions on sleep duration. CI, confidence interval; Chi^2^, chi-square; df, degrees of freedom; I^2^, I-squared; IV, inverse variance; P, *p* value; SD, standard deviation; Tau^2^, between-study variance.

Fourteen trials (*n* = 2,368; intervention, *n* = 1,265; control, *n* = 1,103) reported sleep duration. Lifestyle interventions were associated with slightly longer sleep duration (MD 0.18 h/day, 95% CI 0.01 to 0.35; I^2^ = 68%) (Figure 4).

##### 3.3.1.4 Screen time

Thirty-five trials (*n* = 9,770; intervention, *n* = 5,503; control, *n* = 4,267) reported ST. Lifestyle interventions were associated with a small reduction in ST (MD − 0.33 h/day, 95% CI − 0.53 to −0.13; I^2^ = 95%) (Supplementary Figure 1).

#### 3.3.2 Secondary outcomes

##### 3.3.2.1 Total physical activity

**Figure 5:**
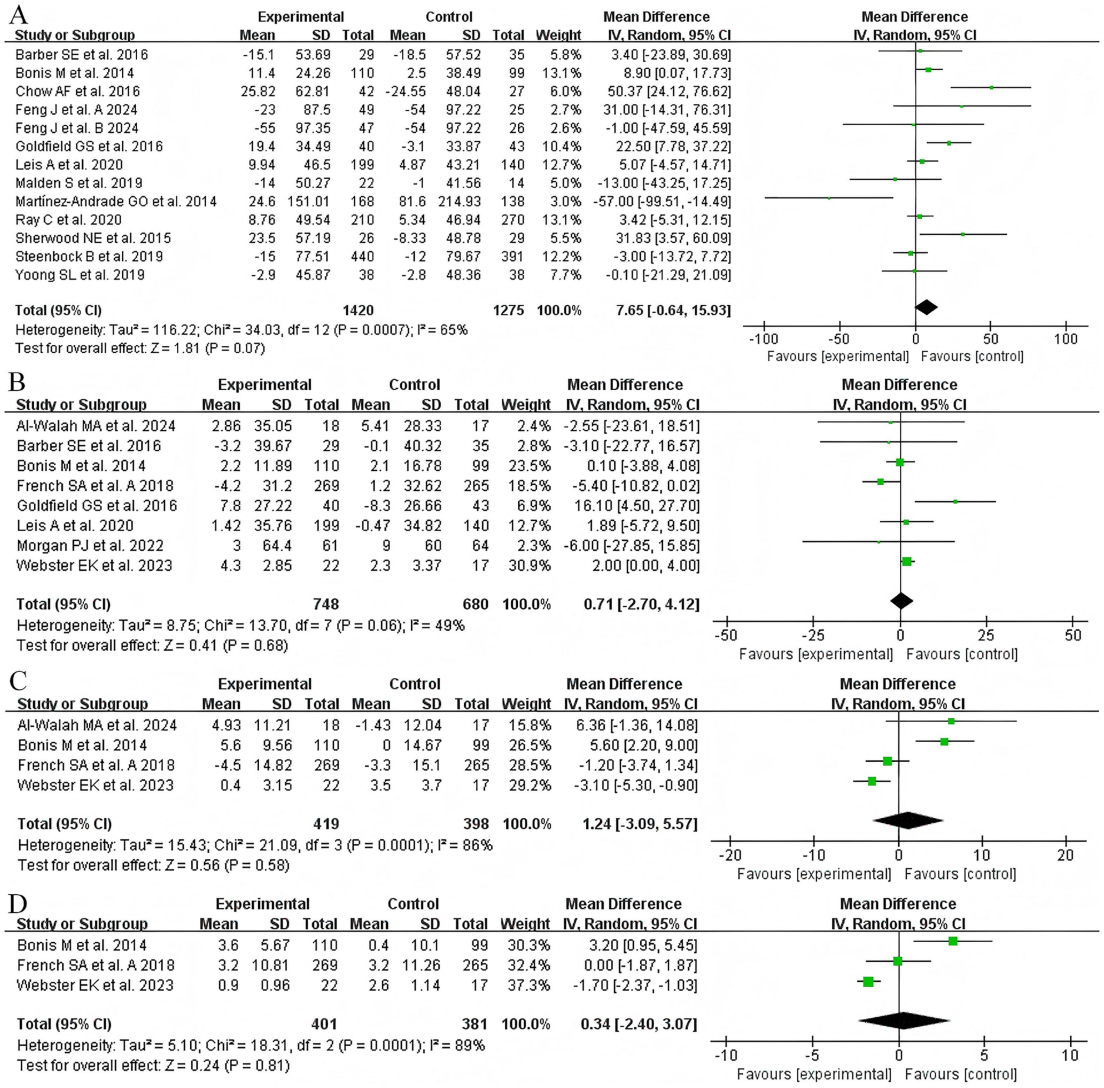
Forest plot of the effects of lifestyle interventions on **(A)** TPA, **(B)** LPA, **(C)** MPA, and **(D)** VPA. CI, confidence interval; Chi^2^, chi-square; df, degrees of freedom; I^2^, I-squared; IV, inverse variance; LPA, light physical activity; MPA, moderate physical activity; *P*, *p* value; SD, standard deviation; Tau^2^, between-study variance; TPA, total physical activity; VPA, vigorous physical activity.

Thirteen trials (*n* = 2,695; intervention, *n* = 1,420; control, *n* = 1,275) reported TPA. The pooled estimate suggested a small increase in TPA, but the 95% CI included the null (MD 7.65 min/day, 95% CI − 0.64 to 15.93; I^2^ = 65%) (Figure 5A).

##### 3.3.2.2 Light physical activity

Eight trials (*n* = 1,428; intervention, *n* = 748; control, *n* = 680) reported LPA. No clear between-group difference was observed (MD 0.71 min/day, 95% CI − 2.70 to 4.12; I^2^ = 49%) (Figure 5B).

##### 3.3.2.3 Moderate physical activity

Three trials (*n* = 817; intervention, *n* = 419; control, *n* = 398) reported MPA. No clear between-group difference was observed (MD 1.24 min/day, 95% CI − 3.09 to 5.57; I^2^ = 86%) (Figure 5C).

##### 3.3.2.4 Vigorous physical activity

Three trials (*n* = 782; intervention, *n* = 401; control, *n* = 381) reported VPA. No clear between-group difference was observed (MD 0.34 min/day, 95% CI − 2.40 to 3.07; I^2^ = 89%) (Figure 5D).

### 3.4 Subgroup analyses

Prespecified subgroup analyses were conducted according to recipient involvement, intervention duration, delivery mode, setting, measurement method, and intervention component; for ST, outcome type was additionally examined. Heterogeneity within subgroup strata varied across outcomes. For LPA, higher heterogeneity was observed in child-involved interventions, interventions lasting >12 weeks, and face-to-face interventions, whereas the corresponding comparison subgroups showed little or no heterogeneity. For MVPA, SB, sleep duration, ST, and TPA, substantial heterogeneity generally persisted across several subgroup strata. However, no statistically significant subgroup differences were detected for most outcomes. For TPA, subgroup differences by measurement method (*p* = 0.063) and intervention duration (*p* = 0.064) approached but did not reach statistical significance. In the additional component-structure subgroup analysis, no statistically significant subgroup differences were observed for MVPA, SB, sleep duration, TPA, or LPA. For ST, the subgroup difference between single-component and multicomponent interventions approached but did not reach statistical significance (*p* = 0.059), suggesting that intervention component structure may partly contribute to variability in ST effects. Overall, these analyses did not provide clear evidence that the prespecified study- and intervention-level characteristics explained the observed between-study heterogeneity (Supplementary Figures 2–7).

### 3.5 Sensitivity analyses

Leave-one-out analyses were conducted to examine the influence of individual trials on the pooled estimates (Supplementary Figure 8). The pooled estimates for MVPA, ST, and sleep duration remained directionally consistent and statistically significant after sequential omission of each study, suggesting that these findings were not driven by any single trial. In contrast, the results for LPA and SB were more sensitive to the exclusion of individual studies. For LPA, omission of one study changed whether the pooled estimate reached statistical significance, whereas for SB, CIs crossed the null in some iterations, although the overall direction of effect remained unchanged. Overall, the sensitivity analyses suggested that the main findings were generally stable, with comparatively lower robustness for LPA and SB.

### 3.6 Publication bias

Funnel plots, together with Egger’s and Begg’s tests, showed no statistically significant evidence of small-study effects for MVPA (Egger’s test, *p* = 0.232; Begg’s test, *p* = 0.194) or TPA (Egger’s test, *p* = 0.783; Begg’s test, *p* = 0.714). For SB, neither test was statistically significant, although Egger’s test approached statistical significance (*p* = 0.053; Begg’s test, *p* = 0.088). For sleep duration, the findings were inconsistent across tests (Egger’s test, *p* = 0.299; Begg’s test, *p* = 0.033). For ST, Begg’s test indicated possible funnel plot asymmetry (*p* < 0.001), whereas Egger’s test was borderline (*p* = 0.078); thus, possible small-study effects could not be ruled out, and the pooled estimate should be interpreted cautiously (Supplementary Figure 9).

### 3.7 Risk of bias of included studies

Across the 43 trials, the overall risk of bias, assessed using RoB 2, was rated as low in 2 trials (4.7%), as having some concerns in 32 trials (74.4%), and as high in 9 trials (20.9%) (Supplementary Figures 10, 11). Common limitations were related to the randomization process and outcome measurement, with additional concerns reflecting the practical challenges of blinding in behavioral interventions.

### 3.8 Certainty of the evidence

Using GRADE, the certainty of the evidence was rated as low for the primary outcomes, mainly because of risk of bias and inconsistency due to heterogeneity (Supplementary Table 6).

## 4 Discussion

This systematic review and meta-analysis of 43 trials involving 13,659 preschool children suggests that lifestyle interventions may be associated with modest improvements in several 24-h movement behaviors. Compared with control conditions, pooled estimates indicated higher MVPA, lower SB, reduced ST, and slightly longer sleep duration, whereas effects on TPA and intensity-specific PA outcomes were imprecise and did not show clear between-group differences. The overall direction of these findings is broadly consistent with previous reviews in related pediatric populations and behavioral domains, including a recent meta-analysis of school-based interventions targeting 24-h movement behaviors in young people, which reported small favorable effects on sedentary behavior-related outcomes and sleep duration, whereas effects on physical activity outcomes were less consistent (26, 27, 44). However, direct comparability remains limited because that review covered a broader age range and focused specifically on school-based delivery, whereas the present review focused on preschool children and included lifestyle interventions delivered across multiple contexts (27). Importantly, the certainty of the evidence for the primary outcomes was low. Only a small proportion of included trials were judged to be at low risk of bias, whereas most were rated as having some concerns or a high risk of bias. Accordingly, even when pooled estimates reached statistical significance, they should be interpreted cautiously as suggesting potentially favorable directions of effect rather than providing robust estimates of intervention efficacy.

Heterogeneity remained substantial for several outcomes, and its sources are likely multifactorial. Although leave-one-out analyses generally supported the direction of the main findings, the SB estimate was less statistically stable in some iterations, indicating greater uncertainty for this outcome. For ST, Begg’s test suggested possible funnel plot asymmetry, whereas Egger’s test was borderline (*p* = 0.078); therefore, small-study effects could not be ruled out. Variability in intervention content, duration, behavioral targets, and comparator conditions may all have contributed to heterogeneity. Differences in outcome measurement are also likely to be important, as the included studies used a mixture of device-based and parent-reported measures, which are not directly equivalent and may differ in validity and susceptibility to bias. Parent-reported measures may capture routine-based behaviors that are difficult to observe continuously, but they are also vulnerable to recall error, proxy-report limitations, and social desirability bias, which may reduce comparability across studies and the precision of pooled estimates (33, 45). By contrast, device-based assessments may better capture continuous movement and sleep–wake patterns, although they are not without limitations (46). This issue may be especially relevant for sleep duration and sedentary- or screen-related outcomes, and recent work in the 24-h movement behavior field has further highlighted the need for caution when interpreting parent-reported compliance patterns (47).

Prespecified subgroup analyses explored whether implementation characteristics, including setting, intervention duration, delivery mode, measurement method, and recipient involvement, were associated with differences in the observed effects. An additional subgroup analysis was conducted according to intervention component structure, comparing single-component and multicomponent interventions. Overall, the subgroup analyses did not identify clear effect modifiers. No statistically significant subgroup differences were detected for MVPA, SB, sleep duration, TPA, or LPA in the component-structure analysis. For ST, the subgroup difference between single-component and multicomponent interventions approached but did not reach statistical significance, suggesting that intervention component structure may partly contribute to variability in ST effects. However, this finding should be interpreted cautiously because heterogeneity remained high in the single-component subgroup. These findings should therefore be regarded as hypothesis-generating rather than confirmatory. This cautious interpretation is consistent with previous work suggesting that structured settings and sufficient intervention exposure may support implementation, while also underscoring that current trial-level evidence remains insufficient to establish these factors as reliable effect modifiers (48, 49). School settings may offer structured schedules, recurring opportunities for activity, teacher facilitation, and access to facilities and equipment, which could improve the consistency of exposure and cumulative intervention dose. In contrast, interventions delivered solely in family or community contexts may be more vulnerable to caregiver time constraints, environmental limitations, and variability in implementation (50, 51).

From pediatric and public health perspectives, even small favorable shifts toward more MVPA, less SB and ST, and slightly longer sleep may be meaningful at the population level if sustained over time (52). However, these effects should be interpreted cautiously in relation to their absolute magnitude. For example, the pooled increase in MVPA (5.77 min/day) and reduction in screen time (0.33 h/day, approximately 20 min/day) were modest and should not be overinterpreted as indicating large individual-level clinical effects. Nevertheless, in the context of current 24-h movement guidelines, such changes may still represent movement in a healthier direction, particularly given the low prevalence of preschool children meeting the combined recommendations. Similarly, the increase in sleep duration (0.18 h/day, approximately 11 min/day) was small at the individual level but may still be relevant if maintained over time and accompanied by broader improvements in daily time use. Adequate sleep is important for growth and neurodevelopment, and shorter sleep during the preschool years has been associated with a higher risk of obesity later in childhood in observational research (53). Persistent sleep problems after age two have also been associated with less favorable neurodevelopmental indicators at school age, suggesting that sleep may play a role in early developmental trajectories (54). Higher PA and lower sedentary and screen-related behaviors have been linked to more favorable cardiometabolic and behavioral profiles in previous research, although the present review did not directly evaluate these downstream health outcomes (55, 56). Overall, the present findings are better interpreted as showing small but potentially relevant behavioral shifts, rather than large or definitively clinically important intervention effects.

An important implication of the 24-h movement framework is that time spent in one behavior necessarily displaces time spent in another within a finite daily time budget (14). This perspective is particularly important given the globally low prevalence of meeting the integrated 24-h movement guidelines across pediatric populations, including during the preschool years, as highlighted by Tapia-Serrano et al. (13). Future studies should therefore examine more explicitly which behaviors are reduced and which are increased when interventions target a specific movement behavior domain, and whether these reallocations contribute to healthier overall 24-h movement profiles (57). Future research should also improve intervention specification and implementation through clearer reporting of intervention content, intended dose, process evaluation, adherence, fidelity, and long-term follow-up. In addition, compositional data analysis and isotemporal substitution models should be incorporated more routinely, as these approaches are better aligned with the finite and co-dependent nature of daily movement behaviors and may more appropriately capture substitution pathways among behaviors. Such methods may help generate more actionable evidence for designing sustainable 24-h movement behavior interventions (58).

## 5 Study limitations

This review has several limitations that should be acknowledged. First, the certainty of the evidence was limited by risk of bias across the included trials. Many trials provided insufficient detail on randomization and allocation concealment. In addition, blinding was often infeasible in behavioral interventions, which may have increased the risk of bias arising from deviations from intended interventions and outcome measurement. Second, intervention reporting was frequently incomplete. Although most interventions were multicomponent, descriptions of core components, intended dose, adherence, and fidelity were often incomplete or inconsistent. This limited mechanistic inference regarding which intervention components may have been effective and whether dose–response relationships were present. It also precluded formal analyses of the relative contribution of individual intervention components. Third, follow-up periods were often short, limiting evaluation of whether behavior changes were maintained or decayed over time. This made it difficult to determine whether small improvements in daily behavior translated into durable behavioral or health benefits. Fourth, heterogeneity was substantial for several outcomes, which reduced the precision and interpretability of the pooled estimates. In addition, possible small-study effects were suggested for ST (Begg’s test, *p* < 0.001; Egger’s test, *p* = 0.078). Accordingly, some pooled estimates may have been overestimated and should be interpreted cautiously.

## 6 Conclusion

This systematic review and meta-analysis suggests that lifestyle interventions may be associated with modest improvements in several 24-h movement behaviors in preschool children. Pooled estimates indicated small increases in MVPA and sleep duration and small reductions in SB and ST. Given the substantial heterogeneity, low certainty of the evidence, and possible small-study effects for ST, these findings are better interpreted as indicating favorable but uncertain directions of effect rather than precise estimates of intervention efficacy. Future research should prioritize high-quality randomized trials with more harmonized outcome definitions, standardized measurement protocols, clearer reporting of intervention content and implementation quality (including intended dose, adherence, and fidelity), and longer-term follow-up. Future studies should also address time reallocation within the 24-h day, for example, by using compositional data analysis and isotemporal substitution approaches, to clarify behavioral substitution pathways and the sustainability of observed effects.

## Data Availability

The original contributions presented in the study are included in the article/Supplementary material, further inquiries can be directed to the corresponding author.
