# Supplementary material for: Lifestyle interventions and 24-hour movement behaviors in preschool children: a systematic review and meta-analysis
Source: Front Public Health. 2026 Jun 17;14:1846736. doi: 10.3389/fpubh.2026.1846736 (PMC13318789; doi:10.3389/fpubh.2026.1846736)
Supplement: Supplementary file 1 [file Data_sheet_1.pdf]

Supplementary Figure 1. Forest plot of the effects of lifestyle interventions on ST.

Study or Subgroup	Experimental Mean	Experimental SD	Experimental Total	Control Mean	Control SD	Control Total	Weight	Mean Difference IV, Random, 95% CI
Byrd-Bredbenner C et al. 2018	-1.6	4.07	85	-0.43	4.36	103	1.6%	-1.17 [-2.38, 0.04]
De Coen V et al. 2012	0	0.9	1032	0.1	1.01	557	3.5%	-0.10 [-0.20, 0.00]
Downing KL et al. 2018	-0.51	1.3	30	0.13	1.17	27	2.6%	-0.64 [-1.28, 0.00]
Feng J et al. A 2024	-0.85	1.58	49	0.11	1.96	25	2.1%	-0.96 [-1.85, -0.07]
Feng J et al. B 2024	-0.69	1.72	47	0.11	1.96	26	2.1%	-0.80 [-1.70, 0.10]
Fitzgibbon ML et al. 2005	-0.5	2.19	143	0	1.45	154	3.0%	-0.50 [-0.93, -0.07]
Fitzgibbon ML et al. 2006	-0.23	1.65	171	-0.06	1.56	153	3.2%	-0.17 [-0.52, 0.18]
Fitzgibbon ML et al. A 2011	-0.4	1.67	96	-0.1	1.68	47	2.7%	-0.30 [-0.89, 0.29]
Fitzgibbon ML et al. A 2013	0.1	1.13	60	0.2	1.22	32	2.9%	-0.10 [-0.61, 0.41]
Fitzgibbon ML et al. B 2011	-0.5	2.46	96	0.5	2.41	47	2.2%	-1.00 [-1.85, -0.15]
Fitzgibbon ML et al. B 2013	0.3	1.47	60	0.2	1.39	32	2.7%	0.10 [-0.51, 0.71]
French SA et al. A 2018	-0.5	1.11	269	-0.4	1.21	132	3.3%	-0.10 [-0.35, 0.15]
French SA et al. B 2018	-0.4	1.61	269	-0.1	1.65	133	3.2%	-0.30 [-0.64, 0.04]
Haines J et al. 2013	-0.81	1.3	55	-0.26	1.75	56	2.7%	-0.55 [-1.12, 0.02]
Haines J et al. 2016	-0.3	1.08	56	-0.3	1.31	56	3.0%	0.00 [-0.44, 0.44]
Hammersley ML et al. 2019	-1.15	2.58	40	-1	1.66	38	2.0%	-0.15 [-1.11, 0.81]
Hinkley T et al. A 2015	-0.65	1.04	12	0.05	0.84	5	2.0%	-0.70 [-1.64, 0.24]
Hinkley T et al. B 2015	-0.17	0.83	12	-0.07	0.76	5	2.2%	-0.10 [-0.92, 0.72]
Kaur N et al. 2024	-0.34	1.53	170	-0.01	1.9	170	3.1%	-0.33 [-0.70, 0.04]
Lerner-Geva L et al. A 2015	-0.1	1	69	0.1	1	34	3.1%	-0.20 [-0.61, 0.21]
Lerner-Geva L et al. B 2015	0.1	1.05	67	0.1	1	34	3.0%	0.00 [-0.42, 0.42]
Lin YM et al. 2021	-1.14	1.39	63	0.17	1.78	66	2.8%	-1.31 [-1.86, -0.76]
Malden S et al. 2019	0.47	1.46	22	0.17	1.19	14	2.1%	0.30 [-0.57, 1.17]
Marsh S et al. 2020	-0.1	0.92	27	0.1	1.06	27	2.8%	-0.20 [-0.73, 0.33]
Martínez-Andrade GO et al. 2014	-0.4	1.45	168	-0.28	1.55	138	3.2%	-0.12 [-0.46, 0.22]
Puder JJ et al. 2011	-0.01	0.95	333	0.21	1.16	292	3.4%	-0.22 [-0.39, -0.05]
Ray C et al. 2020	-0.03	0.71	261	0.07	0.72	325	3.5%	-0.10 [-0.22, 0.02]
Rifas-Shiman SL et al. 2017	-0.5	1.41	253	-0.23	1.25	192	3.3%	-0.27 [-0.52, -0.02]
Steenbock B et al. 2019	0.15	0.84	440	0.15	0.84	391	3.5%	0.00 [-0.11, 0.11]
Taveras EM et al. 2011	-0.54	1.41	253	-0.08	1.33	192	3.3%	-0.46 [-0.72, -0.20]
Tomayko EJ et al. 2018	-0.25	1.8	225	-0.06	2.02	225	3.2%	-0.19 [-0.54, 0.16]
van Grieken A et al. 2014	-0.02	1.14	156	-0.03	1.02	142	3.3%	0.01 [-0.24, 0.26]
Walton K et al. 2016	-0.4	1.06	27	0.1	1.04	21	2.7%	-0.50 [-1.10, 0.10]
Yilmaz G et al. 2015	-1.08	0.3	187	0.13	0.34	176	3.5%	-1.21 [-1.28, -1.14]
Østbye T et al. 2012	-0.23	1.13	200	-0.12	1.13	200	3.4%	-0.11 [-0.33, 0.11]
**Total (95% CI)**			**5503**			**4267**	**100.0%**	**-0.33 [-0.53, -0.13]**

Heterogeneity: $Tau^2 = 0.31$; $Chi^2 = 726.49$, df = 34 ($P < 0.00001$); $I^2 = 95\%$

Test for overall effect: Z = 3.17 (P = 0.002)

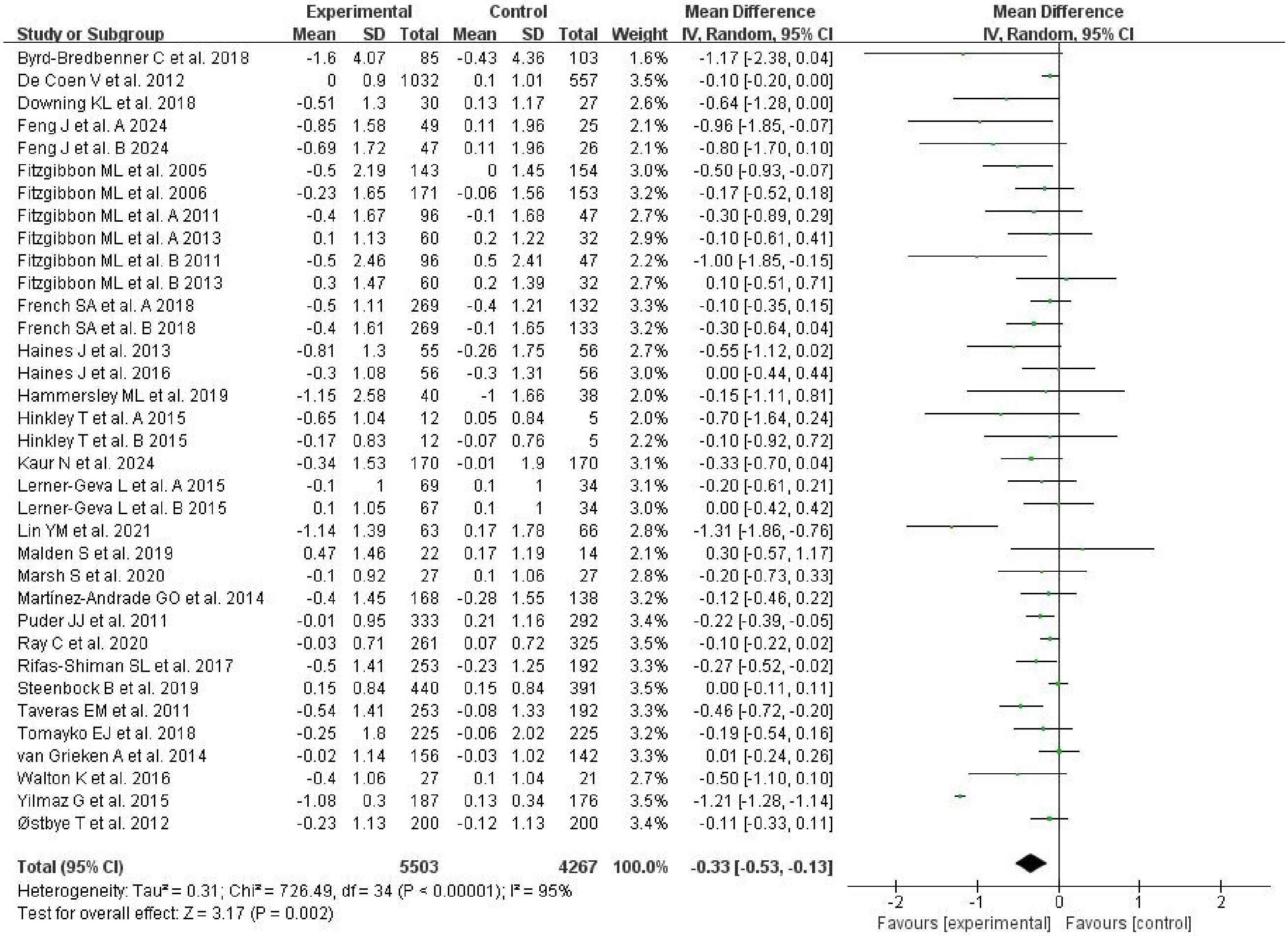

Abbreviations: CI, confidence interval; Chi^2, chi-square; df, degrees of freedom; I^2, I-squared; IV, inverse variance; P, p value; SD, standard deviation; ST, screen time; Tau^2, between-study variance.
