# Supplementary material for: Lifestyle interventions and 24-hour movement behaviors in preschool children: a systematic review and meta-analysis
Source: Front Public Health. 2026 Jun 17;14:1846736. doi: 10.3389/fpubh.2026.1846736 (PMC13318789; doi:10.3389/fpubh.2026.1846736)
Supplement: Supplementary file 2 [file Data_sheet_10.pdf]

Supplementary Figure 6. Subgroup analyses for screen time

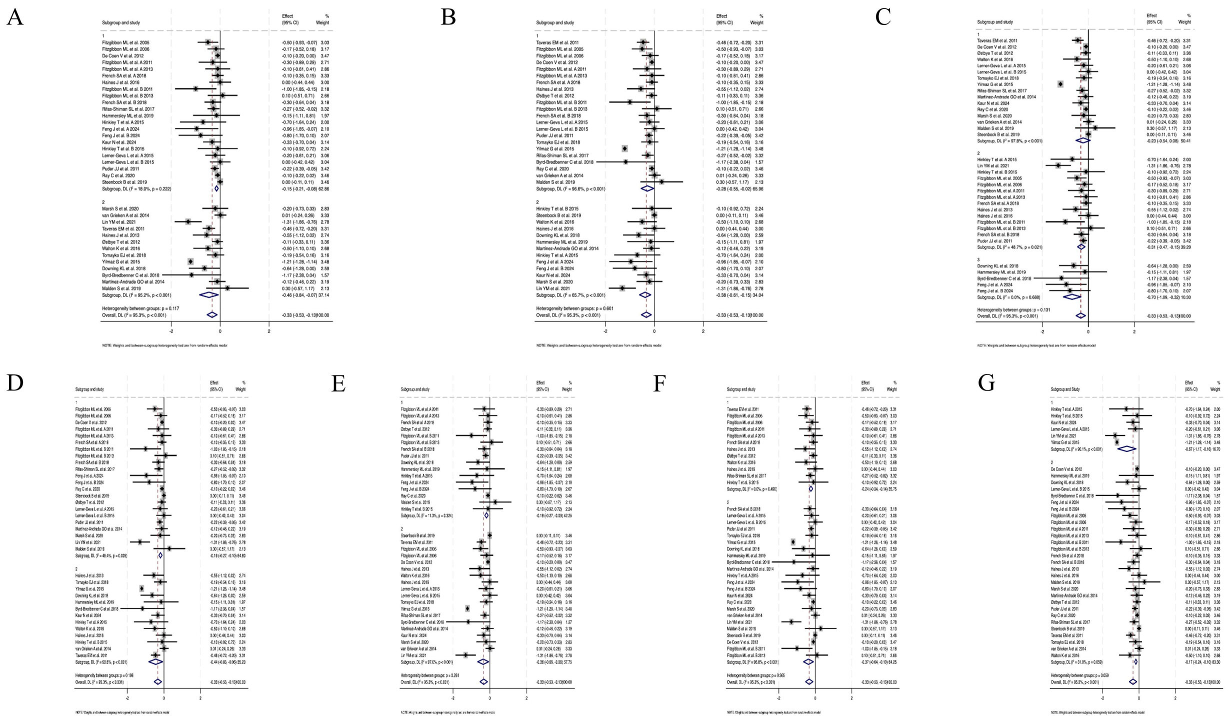

Forest plots showing subgroup analyses for ST by (A) recipient involvement, (B) intervention duration, (C) delivery mode, (D) setting, (E) measurement method, (F) outcome type, and (G) intervention component. In panel A, 1 = children involved and 2 = non-children involved. In panel B, 1 = >12 weeks and 2 = ≤12 weeks. In panel C, 1 = mixed delivery, 2 = FTF delivery, and 3 = online delivery. In panel D, 1 = school involved and 2 = non-school involved. In panel E, 1 = accelerometer and 2 = parent questionnaire. In panel F, 1 = TV only and 2 = total screen time. Effect estimates were pooled using the DerSimonian–Laird random-effects model.

Abbreviations: DL, DerSimonian–Laird; FTF, face-to-face; ST, screen time.
