# Supplementary material for: Lifestyle interventions and 24-hour movement behaviors in preschool children: a systematic review and meta-analysis
Source: Front Public Health. 2026 Jun 17;14:1846736. doi: 10.3389/fpubh.2026.1846736 (PMC13318789; doi:10.3389/fpubh.2026.1846736)
Supplement: Supplementary file 3 [file Data_sheet_11.pdf]

Supplementary Figure 7. Subgroup analyses for total physical activity

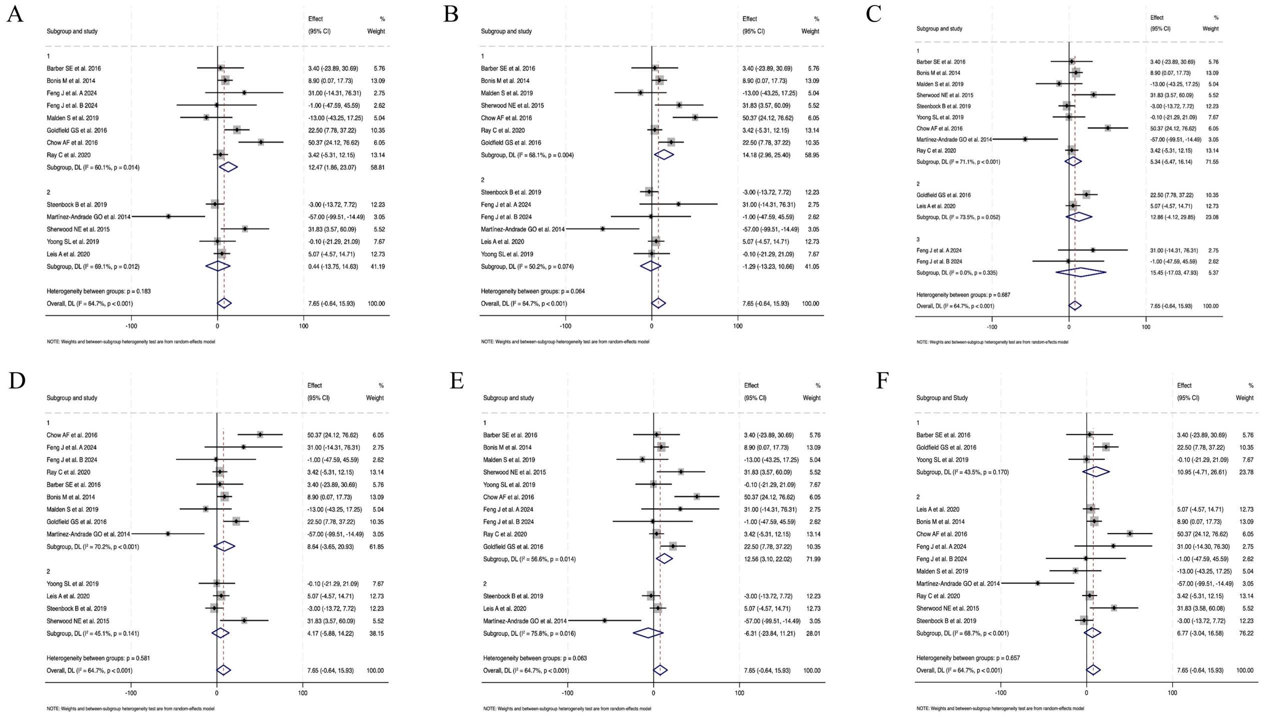

Forest plots showing subgroup analyses for TPA by (A) recipient involvement, (B) intervention duration, (C) delivery mode, (D) setting, (E) measurement method, and (F) intervention component. In panel A, 1 = children involved and 2 = non-children involved. In panel B, 1 = >12 weeks and 2 = ≤12 weeks. In panel C, 1 = mixed delivery, 2 = FTF delivery, and 3 = online delivery. In panel D, 1 = school involved and 2 = non-school involved. In panel E, 1 = accelerometer and 2 = parent questionnaire. Effect estimates were pooled using the DerSimonian–Laird random-effects model.

Abbreviations: DL, DerSimonian–Laird; FTF, face-to-face; TPA, total physical activity.
