# Supplementary material for: Lifestyle interventions and 24-hour movement behaviors in preschool children: a systematic review and meta-analysis
Source: Front Public Health. 2026 Jun 17;14:1846736. doi: 10.3389/fpubh.2026.1846736 (PMC13318789; doi:10.3389/fpubh.2026.1846736)
Supplement: Supplementary file 4 [file Data_sheet_2.pdf]

Supplementary Figure 8. Leave-one-out sensitivity analyses for movement behavior outcomes

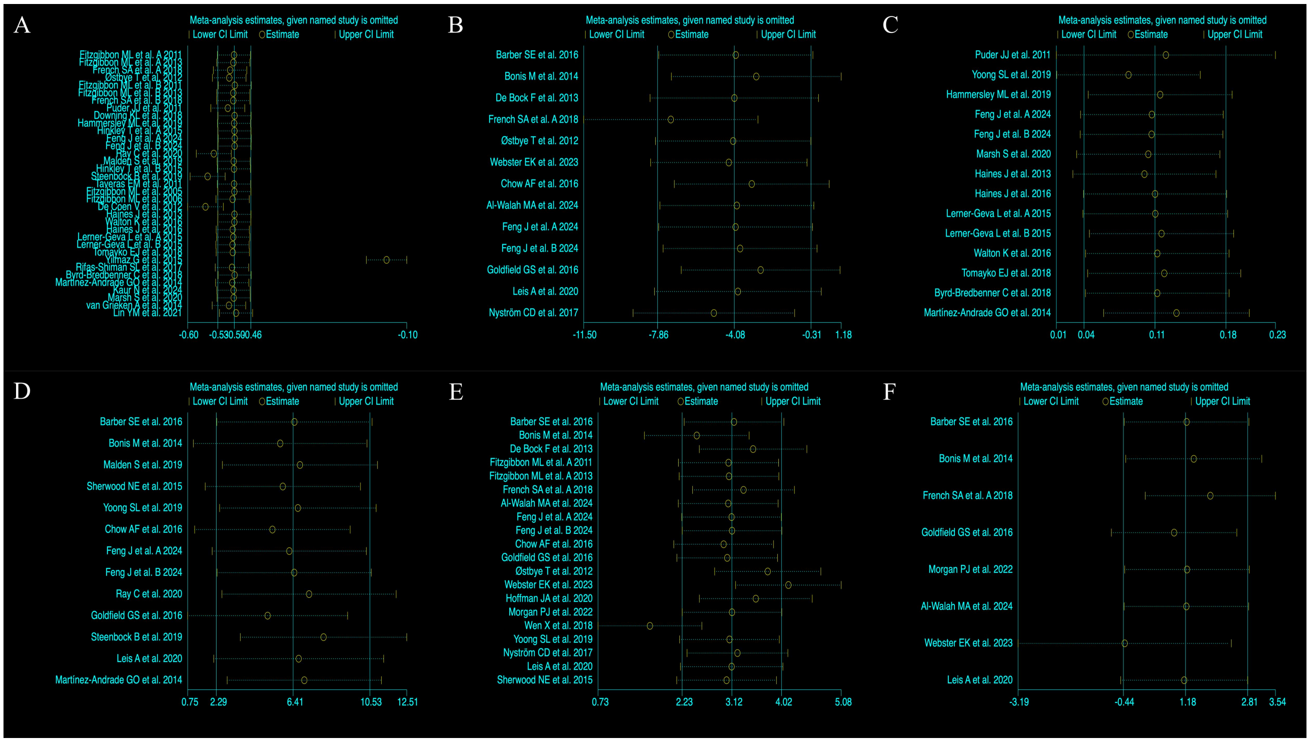

Forest plots showing leave-one-out sensitivity analyses for movement behavior outcomes. Panels show results for (A) ST, (B) SB, (C) sleep duration, (D) TPA, (E) MVPA, and (F) LPA. Each iteration represents the pooled estimate after sequential omission of one study.

Abbreviations: LPA, light physical activity; MVPA, moderate-to-vigorous physical activity; SB, sedentary behavior; ST, screen time; TPA, total physical activity.
