# Supplementary material for: Lifestyle interventions and 24-hour movement behaviors in preschool children: a systematic review and meta-analysis
Source: Front Public Health. 2026 Jun 17;14:1846736. doi: 10.3389/fpubh.2026.1846736 (PMC13318789; doi:10.3389/fpubh.2026.1846736)
Supplement: Supplementary file 5 [file Data_sheet_3.pdf]

Supplementary Figure 9. Funnel plots for movement behavior outcomes

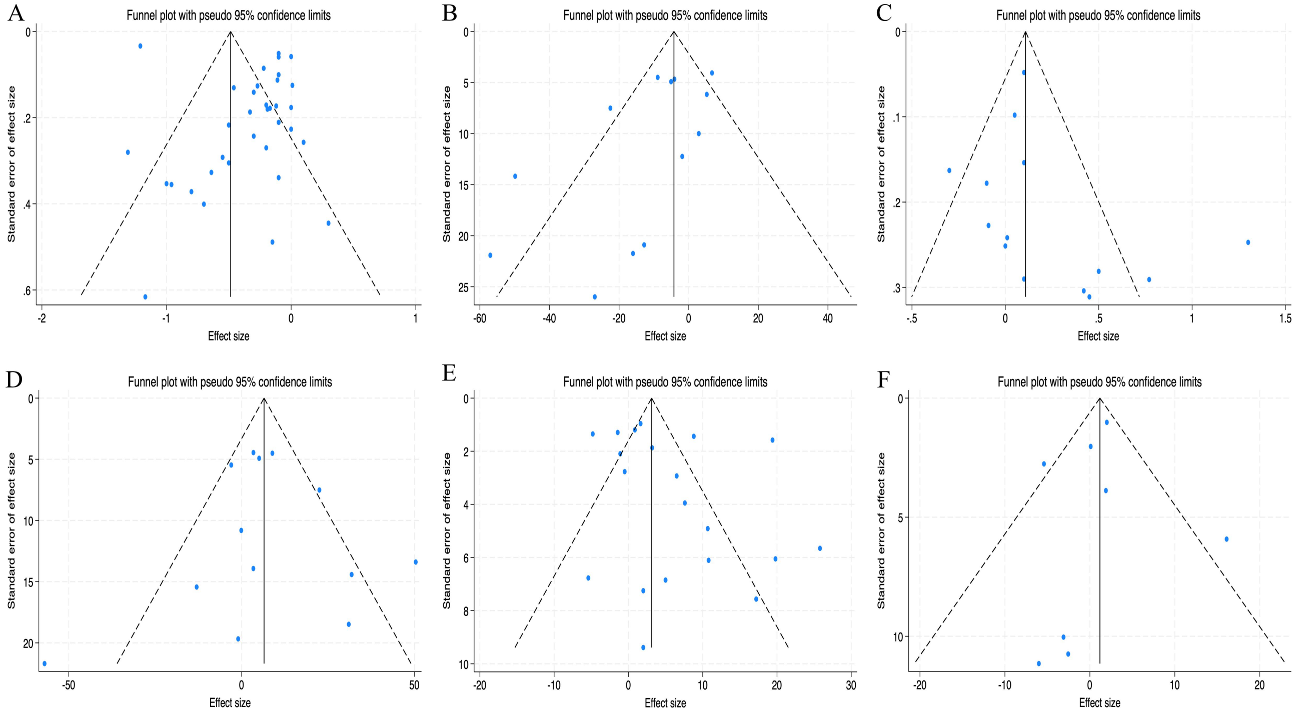

Funnel plots showing publication bias assessments for movement behavior outcomes. Panels show results for (A) ST, (B) SB, (C) sleep duration, (D) TPA, (E) MVPA, and (F) LPA. Egger's and Begg's tests were used to assess funnel plot asymmetry.

Abbreviations: LPA, light physical activity; MVPA, moderate-to-vigorous physical activity; SB, sedentary behavior; ST, screen time; TPA, total physical activity.
