# Supplementary material for: Lifestyle interventions and 24-hour movement behaviors in preschool children: a systematic review and meta-analysis
Source: Front Public Health. 2026 Jun 17;14:1846736. doi: 10.3389/fpubh.2026.1846736 (PMC13318789; doi:10.3389/fpubh.2026.1846736)
Supplement: Supplementary file 6 [file Data_sheet_4.pdf]

Supplementary Figure 10. Domain-level and overall risk-of-bias judgements for included individually randomized trials assessed using RoB 2.

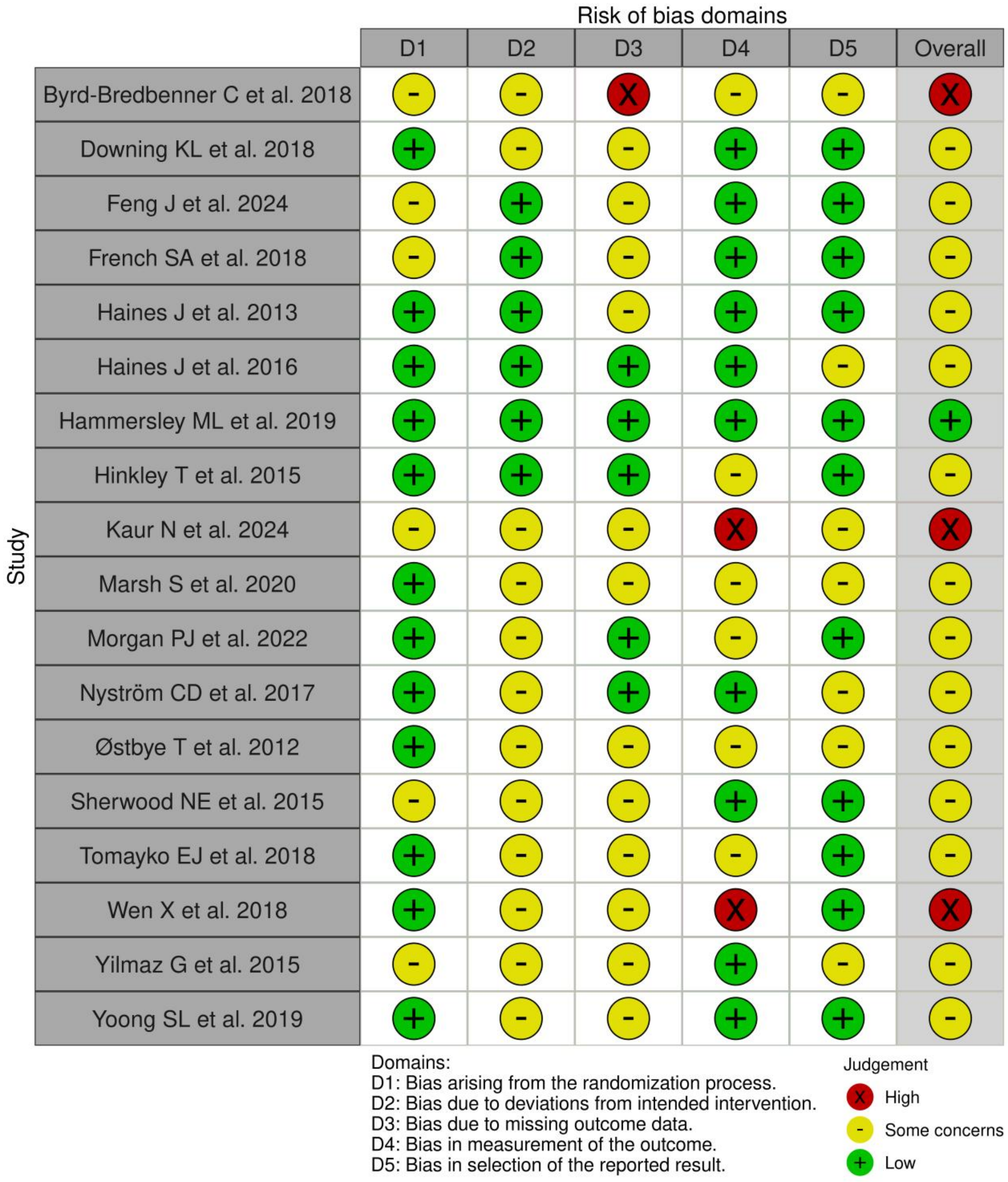


Abbreviations: RoB 2, revised Cochrane risk-of-bias tool for randomized trials.
