# Supplementary material for: Lifestyle interventions and 24-hour movement behaviors in preschool children: a systematic review and meta-analysis
Source: Front Public Health. 2026 Jun 17;14:1846736. doi: 10.3389/fpubh.2026.1846736 (PMC13318789; doi:10.3389/fpubh.2026.1846736)
Supplement: Supplementary file 8 [file Data_sheet_6.pdf]

Supplementary Figure 2. Subgroup analyses for light physical activity

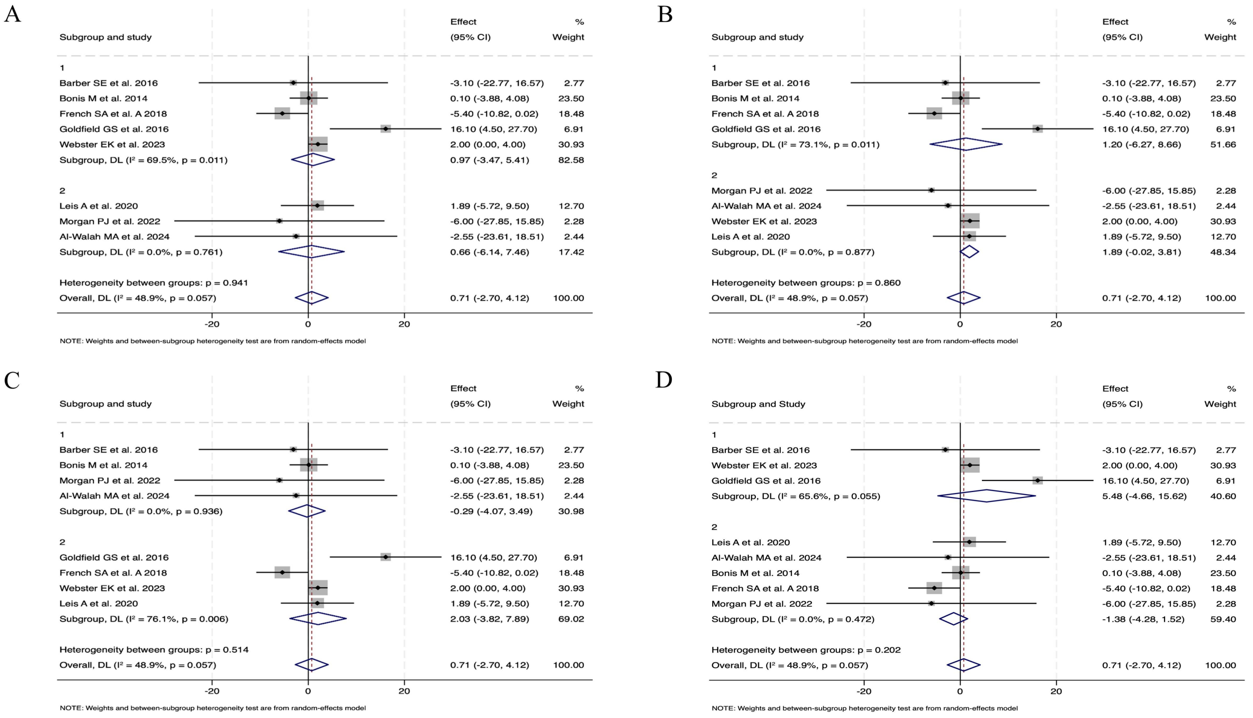

Forest plots showing subgroup analyses for LPA by (A) recipient involvement, (B) intervention duration, (C) delivery mode, and (D) intervention component. In panel A, 1 = children involved and 2 = non-children involved. In panel B, 1 = >12 weeks and 2 = ≤12 weeks. In panel C, 1 = mixed delivery and 2 = FTF delivery. Effect estimates were pooled using the DerSimonian–Laird random-effects model.

Abbreviations: DL, DerSimonian–Laird; FTF, face-to-face; LPA, light physical activity.
