# Supplementary material for: Lifestyle interventions and 24-hour movement behaviors in preschool children: a systematic review and meta-analysis
Source: Front Public Health. 2026 Jun 17;14:1846736. doi: 10.3389/fpubh.2026.1846736 (PMC13318789; doi:10.3389/fpubh.2026.1846736)
Supplement: Supplementary file 9 [file Data_sheet_7.pdf]

frontiers

Supplementary Figure 3. Subgroup analyses for moderate-to-vigorous physical activity

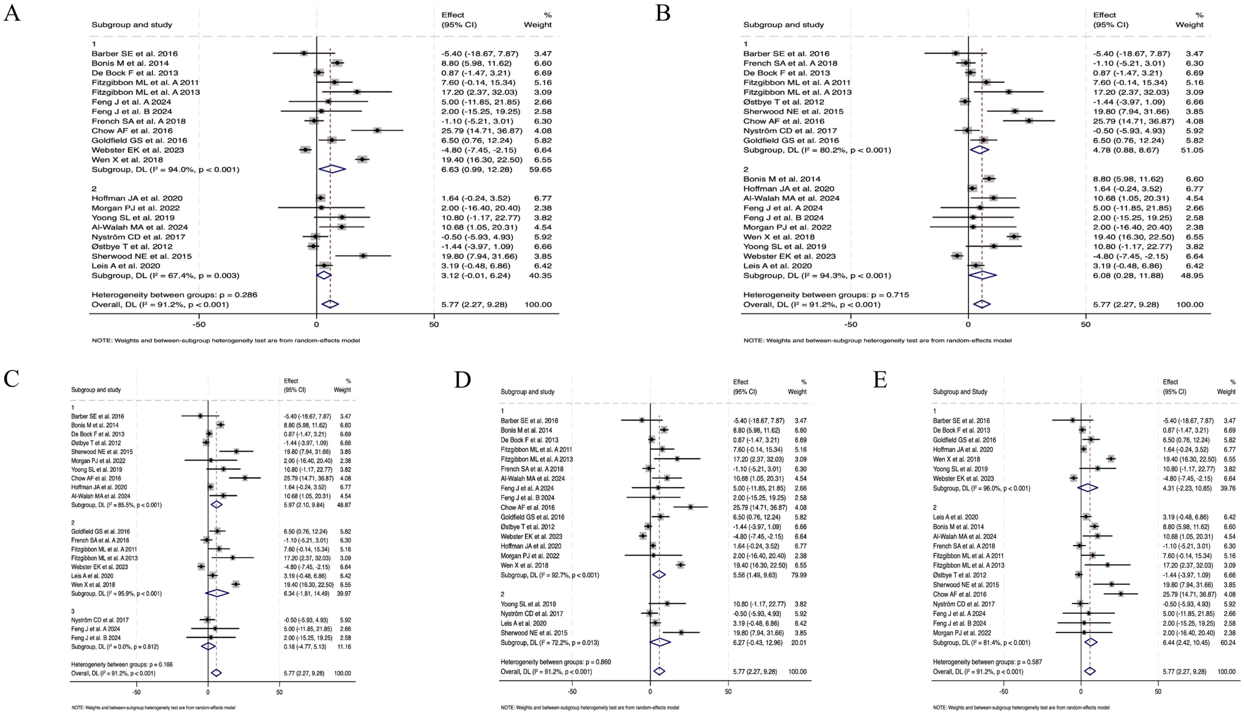

Forest plots showing subgroup analyses for MVPA by (A) recipient involvement, (B) intervention duration, (C) delivery mode, (D) setting, and (E) intervention component. In panel A, 1 = children involved and 2 = non-children involved. In panel B, 1 = >12 weeks and 2 = ≤12 weeks. In panel C, 1 = mixed delivery, 2 = FTF delivery, and 3 = online delivery. In panel D, 1 = school involved and 2 = non-school involved. Effect estimates were pooled using the DerSimonian–Laird random-effects model.

Abbreviations: DL, DerSimonian–Laird; FTF, face-to-face; MVPA, moderate-to-vigorous physical activity.
