# Supplementary material for: Lifestyle interventions and 24-hour movement behaviors in preschool children: a systematic review and meta-analysis
Source: Front Public Health. 2026 Jun 17;14:1846736. doi: 10.3389/fpubh.2026.1846736 (PMC13318789; doi:10.3389/fpubh.2026.1846736)
Supplement: Supplementary file 10 [file Data_sheet_8.pdf]

Supplementary Figure 4. Subgroup analyses for sedentary behavior

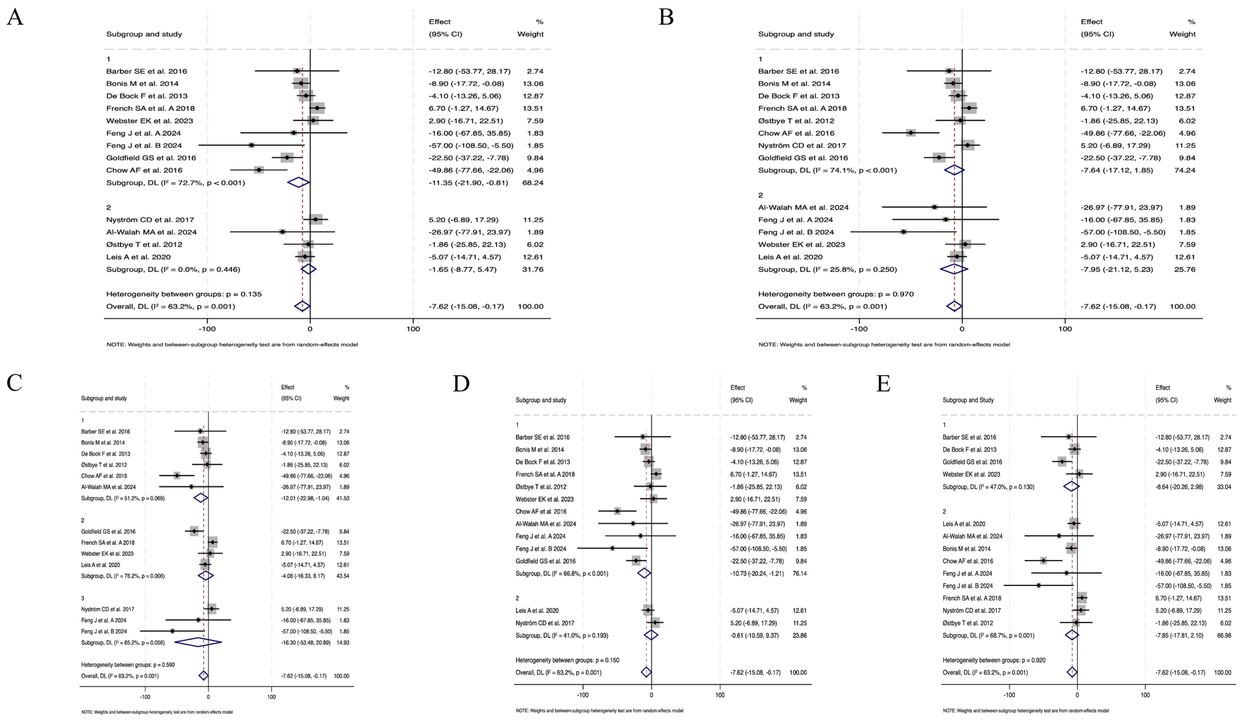

Forest plots showing subgroup analyses for SB by (A) recipient involvement, (B) intervention duration, (C) delivery mode, (D) setting, and (E) intervention component. In panel A, 1 = children involved and 2 = non-children involved. In panel B, 1 = >12 weeks and 2 = ≤12 weeks. In panel C, 1 = mixed delivery, 2 = FTF delivery, and 3 = online delivery. In panel D, 1 = school involved and 2 = non-school involved. Effect estimates were pooled using the DerSimonian–Laird random-effects model.

Abbreviations: DL, DerSimonian–Laird; FTF, face-to-face; SB, sedentary behavior.
