# Supplementary material for: Lifestyle interventions and 24-hour movement behaviors in preschool children: a systematic review and meta-analysis
Source: Front Public Health. 2026 Jun 17;14:1846736. doi: 10.3389/fpubh.2026.1846736 (PMC13318789; doi:10.3389/fpubh.2026.1846736)

**PubMed 3,492**

| Population | "child*"[Title/Abstract] OR "infant*"[Title/Abstract] OR "toddler*"[Title/Abstract] OR "baby"[Title/Abstract] OR "babies"[Title/Abstract] OR "preschool*"[Title/Abstract] OR "kindergarten*"[Title/Abstract] OR "early childhood"[Title/Abstract] OR "early years"[Title/Abstract] OR "childcare"[Title/Abstract] OR "pediatric*"[Title/Abstract] OR "paediatric*"[Title/Abstract] |
| --- | --- |
| interventions | "lifestyle intervention"[Title/Abstract] OR "lifestyle interventions"[Title/Abstract] OR "lifestyle program"[Title/Abstract] OR "lifestyle programs"[Title/Abstract] OR "lifestyle programme"[Title/Abstract] OR "lifestyle programmes"[Title/Abstract] OR "physical activity"[Title/Abstract] OR "exercise"[Title/Abstract] OR "structured physical activity"[Title/Abstract] OR "movement program"[Title/Abstract] OR "movement programme"[Title/Abstract] OR "fitness program"[Title/Abstract] OR "fitness programme"[Title/Abstract] OR "motor skill"[Title/Abstract] OR "motor skills"[Title/Abstract] OR "fundamental movement skill"[Title/Abstract] OR "fundamental movement skills"[Title/Abstract] OR "active play"[Title/Abstract] OR "moderate-to-vigorous physical activity"[Title/Abstract] OR "MVPA"[Title/Abstract] OR "physical education"[Title/Abstract] OR "nutrition"[Title/Abstract] OR "nutrition education"[Title/Abstract] OR "diet"[Title/Abstract] OR "dietary"[Title/Abstract] OR "healthy eating"[Title/Abstract] OR "dietary intake"[Title/Abstract] OR "food intake"[Title/Abstract] OR "diet quality"[Title/Abstract] OR "fruit"[Title/Abstract] OR "fruits"[Title/Abstract] OR "vegetable"[Title/Abstract] OR "vegetables"[Title/Abstract] OR "snack"[Title/Abstract] OR "snacks"[Title/Abstract] OR "sugar-sweetened beverage"[Title/Abstract] OR "energy intake"[Title/Abstract] OR "feeding practice"[Title/Abstract] OR "feeding practices"[Title/Abstract] OR "sedentary"[Title/Abstract] OR "sedentary behavior"[Title/Abstract] OR "sedentary behaviour"[Title/Abstract] OR "sedentary time"[Title/Abstract] OR "sitting"[Title/Abstract] OR "screen time"[Title/Abstract] OR "television viewing"[Title/Abstract] OR "television"[Title/Abstract] OR "TV"[Title/Abstract] OR "media use"[Title/Abstract] OR "electronic media"[Title/Abstract] OR "electronic game"[Title/Abstract] OR "electronic games"[Title/Abstract] OR "video game"[Title/Abstract] OR "video games"[Title/Abstract] OR "computer"[Title/Abstract] OR "computer use"[Title/Abstract] OR "tablet"[Title/Abstract] OR "tablets"[Title/Abstract] OR "iPad"[Title/Abstract] OR "mobile device"[Title/Abstract] OR "mobile devices"[Title/Abstract] OR "smartphone"[Title/Abstract] OR "smartphones"[Title/Abstract] OR "sleep"[Title/Abstract] OR "sleep duration"[Title/Abstract] OR "sleep quality"[Title/Abstract] OR "bedtime"[Title/Abstract] OR "sleep hygiene"[Title/Abstract] OR "sleep routine"[Title/Abstract] OR "sleep routines"[Title/Abstract] OR "sleep education"[Title/Abstract] |
| Outcomes | (("sedentaries"[All Fields] OR "sedentary behavior"[MeSH Terms] OR ("sedentary"[All Fields] AND "behavior"[All Fields]) OR "sedentary behavior"[All Fields] OR "sedentariness"[All Fields] OR "sedentary"[All Fields]) AND "behavio r*"[Title/Abstract]) OR "sedentary lifestyle*"[Title/Abstract] OR "physical inactivit*"[Title/Abstract] OR "sitting"[Title/Abstract] OR "sitting time"[Title/Abstract] OR ("stationar*"[All Fields] AND "behavio r*"[Title/Abstract]) OR "screen time"[Title/Abstract] OR "television viewing"[Title/Abstract] OR "videogame*"[Title/Abstract] OR "video game*"[Title/Abstract] OR "screen-based media"[Title/Abstract] OR "sleep*"[Title/Abstract] OR "bedtime*"[Title/Abstract] OR "sleep timing"[Title/Abstract] OR "insomnia*"[Title/Abstract] OR "polysomnograph*"[Title/Abstract] OR "time in bed"[Title/Abstract] OR "awak*"[Title/Abstract] OR "waking"[Title/Abstract] OR "REM"[Title/Abstract] OR "rapid eye movement"[Title/Abstract] OR "sleep quality"[Title/Abstract] OR "sleep latency"[Title/Abstract] OR "sleep efficiency"[Title/Abstract] OR "sleep duration"[Title/Abstract] OR "sleep hygiene"[Title/Abstract] OR "sleep satisfaction"[Title/Abstract] OR "sleep routine"[Title/Abstract] OR "sleep onset"[Title/Abstract] OR "sleep diary"[Title/Abstract] OR "sleep practice*"[Title/Abstract] OR "sleep habit*"[Title/Abstract] OR "physical activity"[Title/Abstract] OR "MVPA"[Title/Abstract] OR "moderate-to-vigorous physical activity"[Title/Abstract] OR "moderate-to-vigorous physical activity"[Title/Abstract] OR "LPA"[Title/Abstract] OR "light physical activity"[Title/Abstract] OR "TPA"[Title/Abstract] OR "total physical activity"[Title/Abstract] |
| Study design | "random*"[Title/Abstract] OR "rct"[Title/Abstract] OR "clinical trial"[Title/Abstract] OR "intervention study"[Title/Abstract] OR "interventional study"[Title/Abstract] |


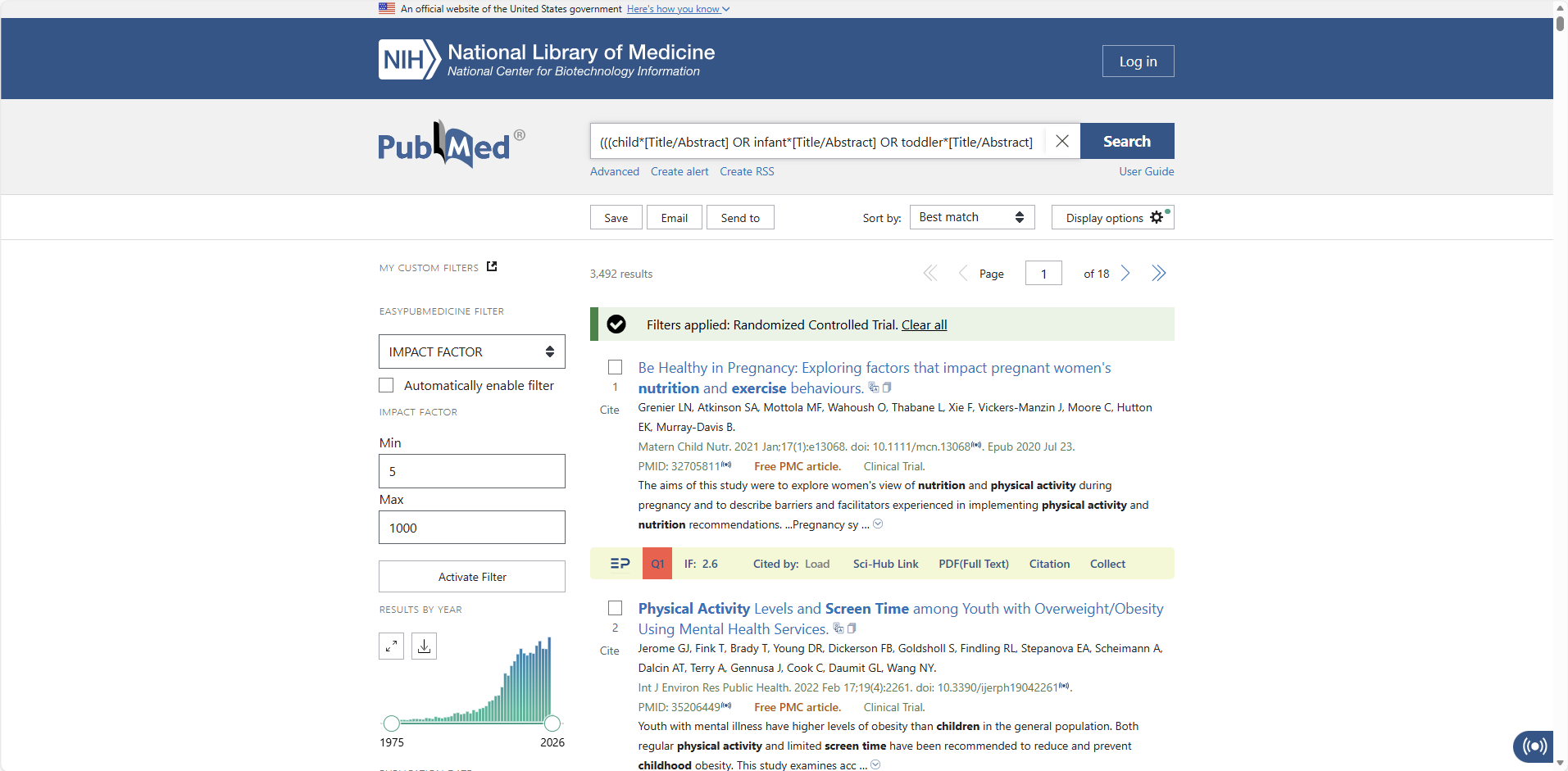


**Scopus 428**

| Population | ( TITLE-ABS-KEY ( child* OR infant* OR toddler* OR baby OR babies OR preschool* OR kindergarten* OR "early childhood" OR "early years" OR childcare OR pediatric* OR paediatric* ) |
| --- | --- |
| interventions | TITLE-ABS-KEY ( "lifestyle intervention" OR "lifestyle interventions" OR "lifestyle program" OR "lifestyle programs" OR "lifestyle programme" OR "lifestyle programmes" OR "physical activity" OR exercise OR "structured physical activity" OR "movement program" OR "movement programme" OR "fitness program" OR "fitness programme" OR "motor skill" OR "motor skills" OR "fundamental movement skill" OR "fundamental movement skills" OR "active play" OR "moderate-to-vigorous physical activity" OR MVPA OR "physical education" OR nutrition OR "nutrition education" OR diet OR dietary OR "healthy eating" OR "dietary intake" OR "food intake" OR "diet quality" OR fruit OR fruits OR vegetable OR vegetables OR snack OR snacks OR "sugar-sweetened beverage" OR "energy intake" OR "feeding practice" OR "feeding practices" OR sedentary OR "sedentary behavior" OR "sedentary behaviour" OR "sedentary time" OR sitting OR "screen time" OR "television viewing" OR television OR TV OR "media use" OR "electronic media" OR "electronic game" OR "electronic games" OR "video game" OR "video games" OR computer OR "computer use" OR tablet OR tablets OR iPad OR "mobile device" OR "mobile devices" OR smartphone OR smartphones OR sleep OR "sleep duration" OR "sleep quality" OR bedtime OR "sleep hygiene" OR "sleep routine" OR "sleep routines" OR "sleep education" ) |
| Outcomes | TITLE-ABS-KEY ( sedentary behavio?r* OR sedentary lifestyle* OR physical inactivit* OR sitting OR "sitting time" OR stationar* behavio?r* OR "screen time" OR "television viewing" OR videogame* OR "video game*" OR "screen-based media" OR sleep* OR bedtime* OR "sleep timing" OR insomnia* OR polysomnograph* OR "time in bed" OR awak* OR waking OR REM OR "rapid eye movement" OR "sleep quality" OR "sleep latency" OR "sleep efficiency" OR "sleep duration" OR "sleep hygiene" OR "sleep satisfaction" OR "sleep routine" OR "sleep onset" OR "sleep diary" OR "sleep practice*" OR "sleep habit*" OR "physical activity" OR MVPA OR "moderate-to-vigorous physical activity" OR "moderate to vigorous physical activity" OR LPA OR "light physical activity" OR TPA OR "total physical activity" ) |
| Study design | TITLE-ABS-KEY ( random* OR rct OR "clinical trial" OR "intervention study" OR "interventional study" ) |


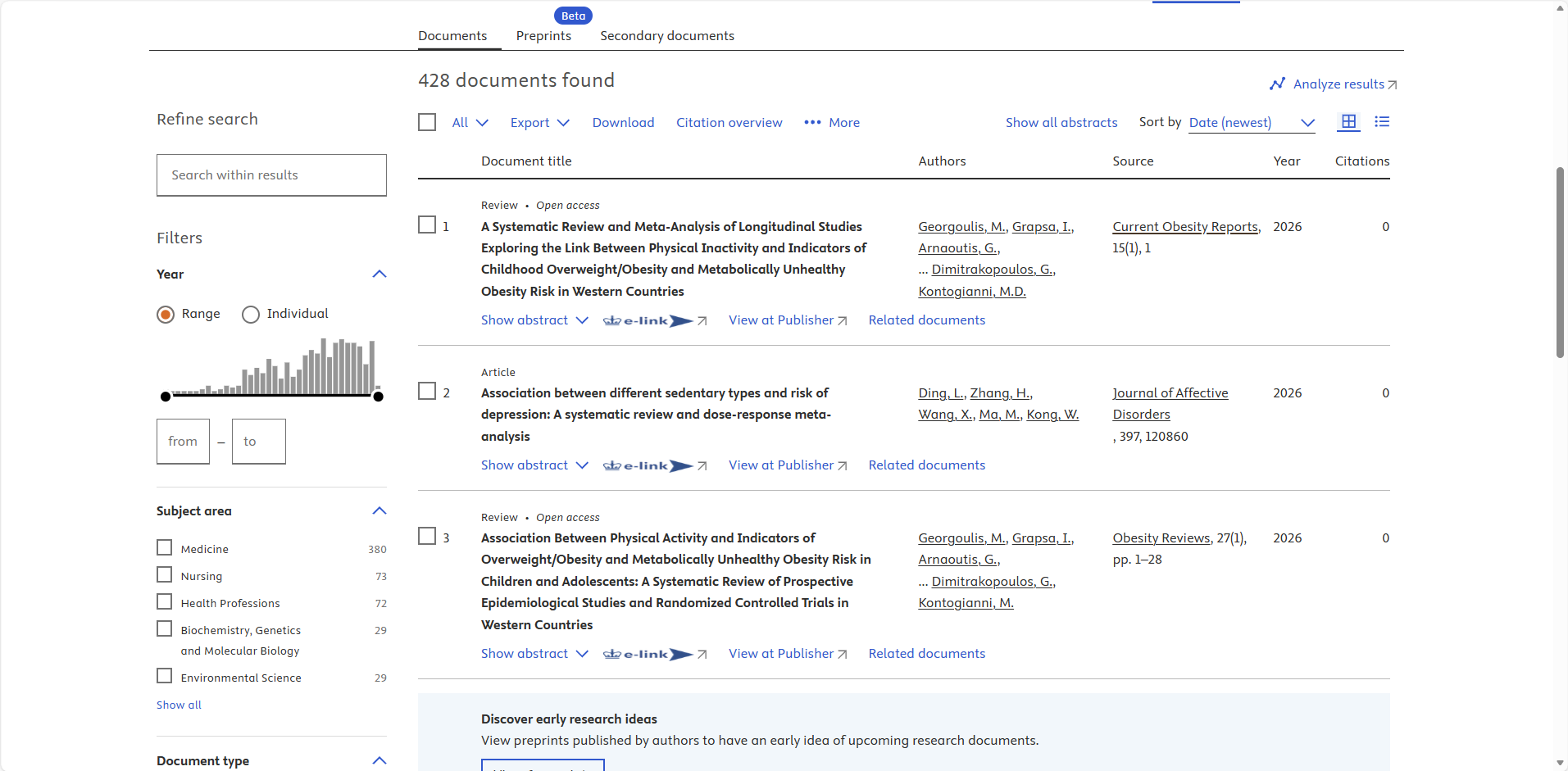


**Web of Science 13,479**

| Population | child* OR infant* OR toddler* OR baby OR babies OR preschool* OR kindergarten* OR "early childhood" OR "early years" OR childcare OR pediatric* OR paediatric* (Topic) |
| --- | --- |
| interventions | "lifestyle intervention" OR "lifestyle interventions" OR "lifestyle program" OR "lifestyle programs" OR "lifestyle programme" OR "lifestyle programmes" OR "physical activity" OR exercise OR "structured physical activity" OR "movement program" OR "movement programme" OR "fitness program" OR "fitness programme" OR "motor skill" OR "motor skills" OR "fundamental movement skill" OR "fundamental movement skills" OR "active play" OR "moderate-to-vigorous physical activity" OR MVPA OR "physical education" OR nutrition OR "nutrition education" OR diet OR dietary OR "healthy eating" OR "dietary intake" OR "food intake" OR "diet quality" OR fruit OR fruits OR vegetable OR vegetables OR snack OR snacks OR "sugar-sweetened beverage" OR "energy intake" OR "feeding practice" OR "feeding practices" OR sedentary OR "sedentary behavior" OR "sedentary behaviour" OR "sedentary time" OR sitting OR "screen time" OR "television viewing" OR television OR TV OR "media use" OR "electronic media" OR "electronic game" OR "electronic games" OR "video game" OR "video games" OR computer OR "computer use" OR tablet OR tablets OR iPad OR "mobile device" OR "mobile devices" OR smartphone OR smartphones OR sleep OR "sleep duration" OR "sleep quality" OR bedtime OR "sleep hygiene" OR "sleep routine" OR "sleep routines" OR "sleep education" (Topic) |
| Outcomes | sedentary behavio?r* OR sedentary lifestyle* OR physical inactivit* OR sitting OR "sitting time" OR stationar* behavio?r* OR "screen time" OR "television viewing" OR videogame* OR "video game*" OR "screen-based media" OR sleep* OR bedtime* OR "sleep timing" OR insomnia* OR polysomnograph* OR "time in bed" OR awak* OR waking OR REM OR "rapid eye movement" OR "sleep quality" OR "sleep latency" OR "sleep efficiency" OR "sleep duration" OR "sleep hygiene" OR "sleep satisfaction" OR "sleep routine" OR "sleep onset" OR "sleep diary" OR "sleep practice*" OR "sleep habit*" OR "physical activity" OR MVPA OR "moderate-to-vigorous physical activity" OR "moderate to vigorous physical activity" OR LPA OR "light physical activity" OR TPA OR "total physical activity" (Topic) |
| Study design | random* OR rct OR "clinical trial" OR "intervention study" OR "interventional study" (Topic) |


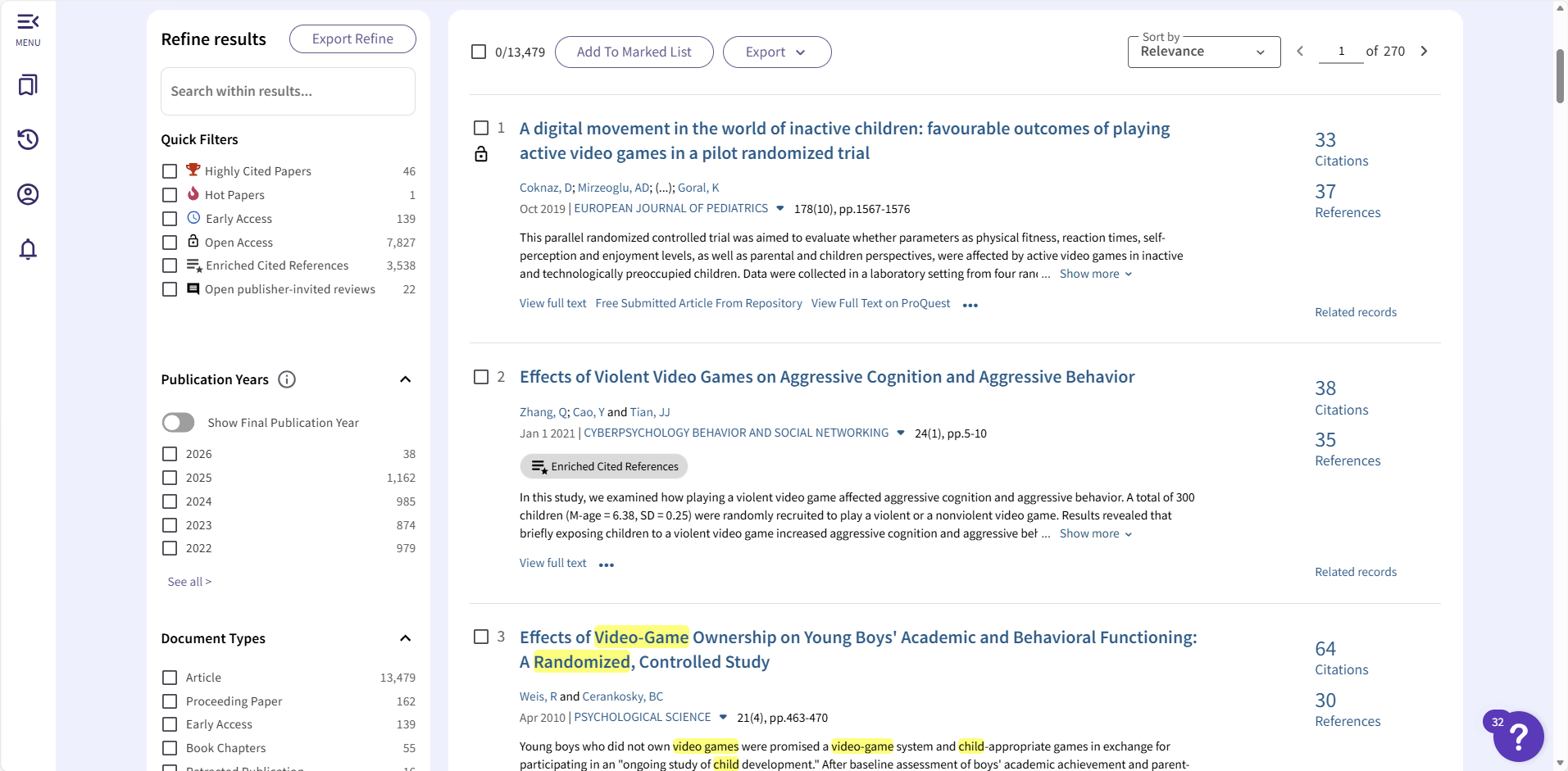


**Cochrane 5,990**

| Population | (Child OR "young child" OR infant OR baby OR babies OR toddler OR "early childhood" OR "early years" OR preschool OR "preschool child" OR "preschool children" OR preschooler OR "pre-school"):ti,ab,kw |
| --- | --- |
| interventions | ("lifestyle intervention" OR "lifestyle interventions" OR "lifestyle program" OR "lifestyle programs" OR "lifestyle programme" OR "lifestyle programmes" OR "physical activity" OR exercise OR "structured physical activity" OR "movement program" OR "movement programme" OR "fitness program" OR "fitness programme" OR "motor skill" OR "motor skills" OR "fundamental movement skill" OR "fundamental movement skills" OR "active play" OR "moderate-to-vigorous physical activity" OR MVPA OR "physical education" OR nutrition OR "nutrition education" OR diet OR dietary OR "healthy eating" OR "dietary intake" OR "food intake" OR "diet quality" OR fruit OR fruits OR vegetable OR vegetables OR snack OR snacks OR "sugar-sweetened beverage" OR "energy intake" OR "feeding practice" OR "feeding practices" OR sedentary OR "sedentary behavior" OR "sedentary behaviour" OR "sedentary time" OR sitting OR "screen time" OR "television viewing" OR television OR TV OR "media use" OR "electronic media" OR "electronic game" OR "electronic games" OR "video game" OR "video games" OR computer OR "computer use" OR tablet OR tablets OR iPad OR "mobile device" OR "mobile devices" OR smartphone OR smartphones OR sleep OR "sleep duration" OR "sleep quality" OR bedtime OR "sleep hygiene" OR "sleep routine" OR "sleep routines" OR "sleep education"):ti,ab,kw |
| Outcomes | (sedentary lifestyle* OR physical inactivit* OR sitting OR "sitting time" OR "screen time" OR "television viewing" OR videogame* OR "video game*" OR "screen-based media" OR sleep* OR bedtime* OR "sleep timing" OR insomnia* OR polysomnograph* OR "time in bed" OR awak* OR waking OR REM OR "rapid eye movement" OR "sleep quality" OR "sleep latency" OR "sleep efficiency" OR "sleep duration" OR "sleep hygiene" OR "sleep satisfaction" OR "sleep routine" OR "sleep onset" OR "sleep diary" OR "sleep practice*" OR "sleep habit*" OR "physical activity" OR MVPA OR "moderate-to-vigorous physical activity" OR "moderate to vigorous physical activity" OR LPA OR "light physical activity" OR TPA OR "total physical activity"):ti,ab,kw |
| Study design | (random* OR rct OR "clinical trial" OR "intervention study" OR "interventional study"):ti,ab,kw |


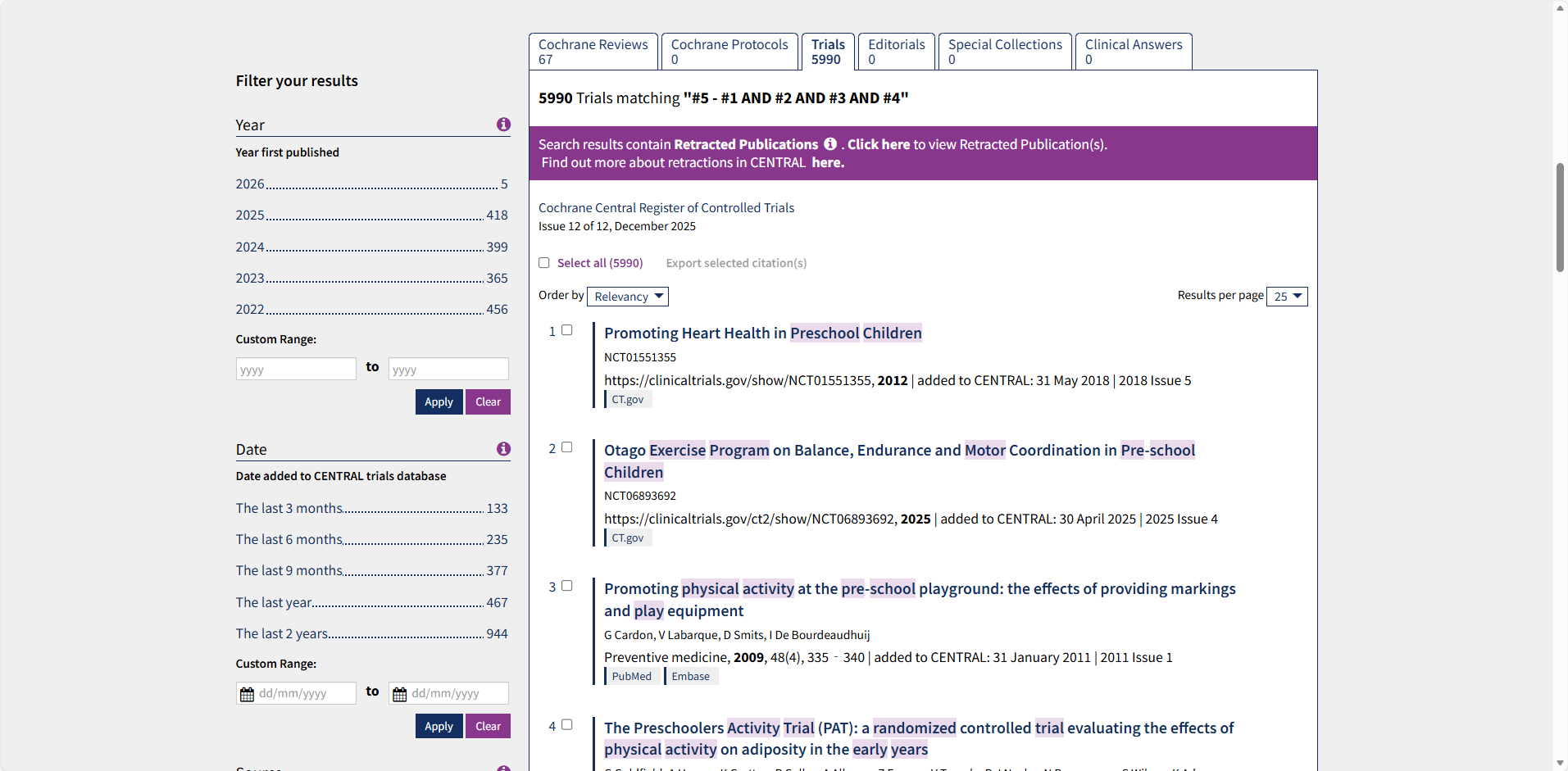


**SPORTDiscus 770**

| Population | XB (child* OR infant* OR toddler* OR baby OR babies OR preschool* OR kindergarten* OR "early childhood" OR "early years" OR childcare OR pediatric* OR paediatric*) |
| --- | --- |
| interventions | XB ("lifestyle intervention" OR "lifestyle interventions" OR "lifestyle program" OR "lifestyle programs" OR "lifestyle programme" OR "lifestyle programmes" OR "physical activity" OR exercise OR "structured physical activity" OR "movement program" OR "movement programme" OR "fitness program" OR "fitness programme" OR "motor skill" OR "motor skills" OR "fundamental movement skill" OR "fundamental movement skills" OR "active play" OR "moderate-to-vigorous physical activity" OR MVPA OR "physical education" OR nutrition OR "nutrition education" OR diet OR dietary OR "healthy eating" OR "dietary intake" OR "food intake" OR "diet quality" OR fruit OR fruits OR vegetable OR vegetables OR snack OR snacks OR "sugar-sweetened beverage" OR "energy intake" OR "feeding practice" OR "feeding practices" OR sedentary OR "sedentary behavior" OR "sedentary behaviour" OR "sedentary time" OR sitting OR "screen time" OR "television viewing" OR television OR TV OR "media use" OR "electronic media" OR "electronic game" OR "electronic games" OR "video game" OR "video games" OR computer OR "computer use" OR tablet OR tablets OR iPad OR "mobile device" OR "mobile devices" OR smartphone OR smartphones OR sleep OR "sleep duration" OR "sleep quality" OR bedtime OR "sleep hygiene" OR "sleep routine" OR "sleep routines" OR "sleep education") |
| Outcomes | XB (sedentary behavio?r* OR sedentary lifestyle* OR physical inactivit* OR sitting OR "sitting time" OR stationar* behavio?r* OR "screen time" OR "television viewing" OR videogame* OR "video game*" OR "screen-based media" OR sleep* OR bedtime* OR "sleep timing" OR insomnia* OR polysomnograph* OR "time in bed" OR awak* OR waking OR REM OR "rapid eye movement" OR "sleep quality" OR "sleep latency" OR "sleep efficiency" OR "sleep duration" OR "sleep hygiene" OR "sleep satisfaction" OR "sleep routine" OR "sleep onset" OR "sleep diary" OR "sleep practice*" OR "sleep habit*" OR "physical activity" OR MVPA OR "moderate-to-vigorous physical activity" OR "moderate to vigorous physical activity" OR LPA OR "light physical activity" OR TPA OR "total physical activity") |
| Study design | XB (random* OR rct OR "clinical trial" OR "intervention study" OR "interventional study") |


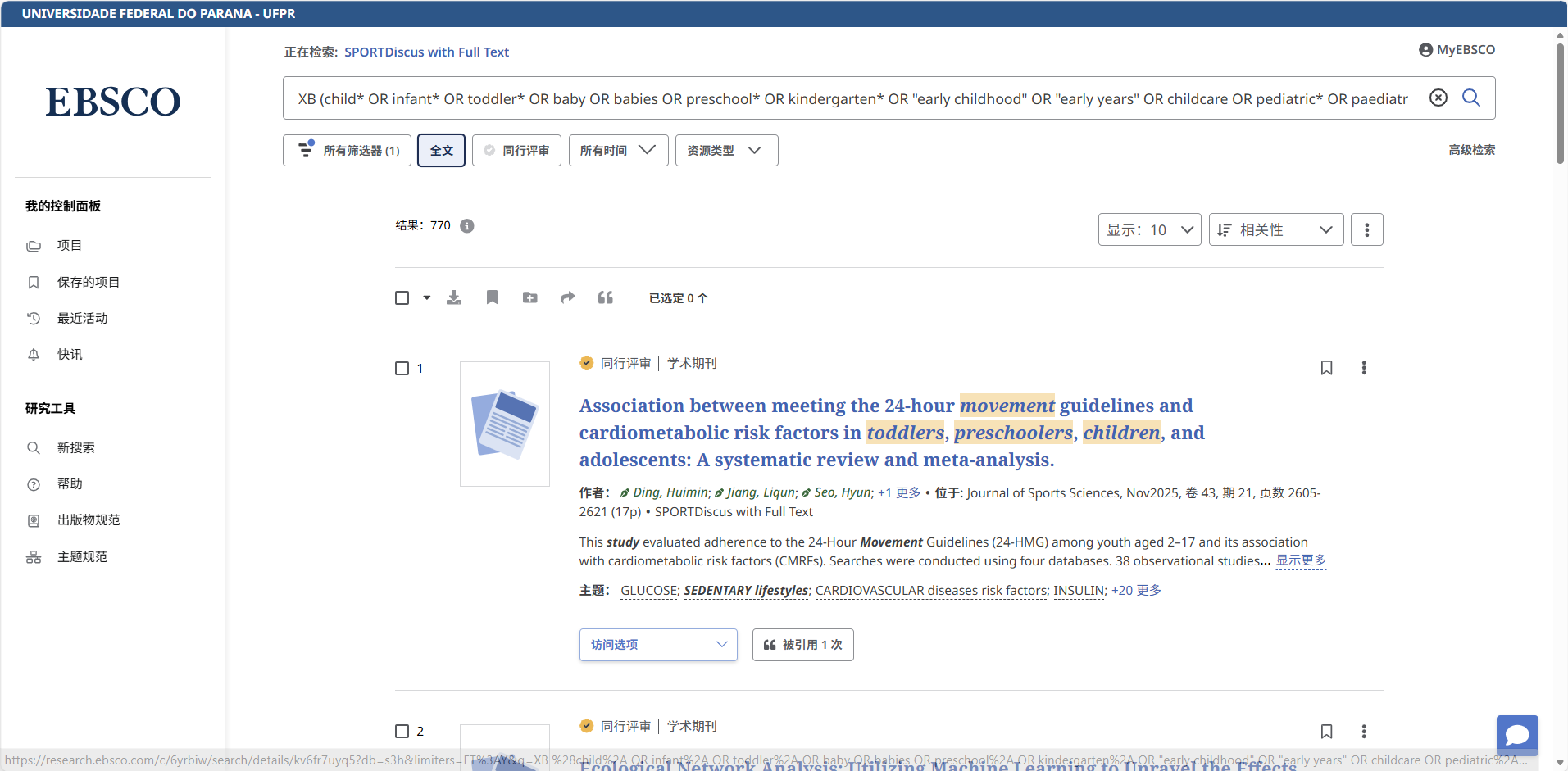

Supplement: Supplementary file 15 [file Table_4.docx]
