# Supplementary material for: Lifestyle interventions and 24-hour movement behaviors in preschool children: a systematic review and meta-analysis
Source: Front Public Health. 2026 Jun 17;14:1846736. doi: 10.3389/fpubh.2026.1846736 (PMC13318789; doi:10.3389/fpubh.2026.1846736)
Supplement: Supplementary file 16 [file Table_5.docx]

**Supplementary Table 5. Characteristics of the included studies**

| **Study** | **Country** | **Study design** | **Trial registration** | **Sample size** | **Sex (female, %)** | **Age, mean (SD), years** | **Intervention** | **Intervention duration** | **Control** | **Measurement tool** | **Theoretical framework** | **Outcomes** |
| --- | --- | --- | --- | --- | --- | --- | --- | --- | --- | --- | --- | --- |
| 1. Al-Walah MA et al. 2024 | Saudi Arabia | CRCT | NR | T = 52; I = 27; C = 25 | I = 48.1%; C = 40.0% | I = 4.18 (0.42); C = 4.16 (0.49) | PA + nutrition lifestyle intervention | 10 weeks | Usual care | ActiGraph GT3X | NR | SB, LPA, MPA, MVPA |
| 1. Barber SE et al. 2016 | United Kingdom | CRCT | NR | T = 64; I = 29; C = 35 | I = 52.4%; C = 52.5% | I = 2.8 (0.7); C = 2.8 (0.8) | PA – focused lifestyle intervention | 12 months | Usual care | ActiGraph GT3X+ | NR | TPA, LPA, MVPA, SB |
| 1. Bonis M et al. 2014 | United States | CRCT | NR | T = 209; I = 110; C = 99 | I = 50.0%; C = 50.5% | I = 3.81 (0.75); C = 3.90 (0.85) | PA + nutrition lifestyle intervention | 6 months | Usual care | ActiGraph GT3X+ | NR | TPA, LPA, VPA, MPA, MVPA, SB |
| 1. Byrd-Bredbenner C et al. 2018 | United States | RCT | NR | T = 489; I = 252; C = 237 | T = 48.0% | T = 3.85 (1.05) | Multicomponent lifestyle intervention | 12 months | Attention control | Parent-reported questionnaire | Socioecological Model | Sleep duration, ST |
| 1. Chow AF et al. 2016 | Canada | CRCT | NR | T = 69; I = 42; C = 27 | I = 39.0%; C = 33.0% | T = 4.75 (NR) | Multicomponent lifestyle intervention | 48 weeks | Wait-list control | Actical accelerometer | McLeroy’s Ecological Model + Population Health Approach | SB, TPA, MVPA |
| 1. De Bock F et al. 2013 | Germany | CRCT | NR | T = 809; I = 433; C = 376 | T = 48.0% | T = 5.0 (0.2) | PA – focused lifestyle intervention | 12 months | Usual care | Actiheart | General Systems Theory | MVPA, SB |
| 1. De Coen V et al. 2012 | Belgium | CRCT | NR | T = 1589; I = 1032; C = 557 | NR | T = 5.0 (0.2) | Multicomponent lifestyle intervention | 24 months | Usual care | Parent-reported questionnaire | Socioecological Model | ST |
| 1. Downing KL et al. 2018 | Australia | RCT | ACTRN12616000628448 | T = 57; I = 30; C = 27 | I = 50.0%; C = 41.0% | I = 3.2 (0.8); C = 2.9 (0.7) | SB – and ST – focused lifestyle intervention | 6 weeks | Wait-list control | activPAL | Social Cognitive Theory + CALO-RE Taxonomy of Behavior Change Techniques | ST |
| 1. Feng J et al. 2024 | China | RCT | NR | T = 147; IA = 49; IB = 47; C = 51 | IA = 59.2%; IB = 48.9%; C = 60.8% | IA = 5.0 (1.0); IB = 4.9 (0.9); C = 4.6 (0.9) | Multicomponent lifestyle intervention | 12 weeks | Wait-list control | ActiGraph wGT3X-BT | NR | TPA, MVPA, SB, sleep duration, ST |
| 1. Fitzgibbon ML et al. 2005 | United States | CRCT | NR | T = 409; I = 197; C = 212 | I = 49.7%; C = 50.5% | I = 4.05 (0.63); C = 4.23 (0.53) | Multicomponent lifestyle intervention | 14 weeks | Usual care | Parent-reported questionnaire | NR | ST |
| 1. Fitzgibbon ML et al. 2006 | United States | CRCT | NR | T = 401; I = 202; C = 199 | I = 47.5%; C = 51.3% | I = 4.23 (NR); C = 4.25 (NR) | Multicomponent lifestyle intervention | 14 weeks | Usual care | Parent-reported questionnaire | Social Cognitive Theory + Self-Determination Theory | ST |
| 1. Fitzgibbon ML et al. 2011 | United States | CRCT | NR | T = 618; I = 325; C = 293 | I = 52.0%; C = 55.0% | I = 4.23 (NR); C = 4.33 (NR) | Multicomponent lifestyle intervention | 14 weeks | Usual care | ActiGraph GT1M | Social Cognitive Theory + Self-Determination Theory | ST, MVPA |
| 1. Fitzgibbon ML et al. 2013 | United States | CRCT | NR | T = 146; I = 72; C = 74 | I = 53.0%; C = 47.0% | I = 4.48 (NR); C = 4.56 (NR) | Multicomponent lifestyle intervention | 14 weeks | Usual care | ActiGraph GT1M | Social Cognitive Theory + Self-Determination Theory | ST, MVPA |
| 1. French SA et al. 2018 | United States | RCT | NR | T = 534; I = 265; C = 269 | I = 50.9%; C = 50.9% | Range: 2–4 | Multicomponent lifestyle intervention | 36 months | Usual care | ActiGraph GT3X+ | Social Ecological Theory | LPA, MPA, MVPA, VPA, SB, ST |
| 1. Goldfield GS et al. 2016 | Canada | CRCT | NCT02293278 | T = 83; I = 43; C = 40 | I = 55.0%; C = 46.5% | I = 3.3 (0.6); C = 3.3 (0.6) | PA – focused lifestyle intervention | 6 months | Usual care | Actical accelerometer | NR | TPA, MVPA, LPA, SB |
| 1. Haines J et al. 2013 | United States | RCT | NR | T = 121; I = 62; C = 59 | I = 43.6%; C = 51.8% | I = 4.1 (1.1); C = 4.0 (1.1) | Multicomponent lifestyle intervention | 4 months | Attention control | Parent-reported questionnaire | NR | Sleep duration, ST |
| 1. Haines J et al. 2016 | United States | RCT | NR | T = 112; I = 56; C = 56 | I = 51.8%; C = 48.2% | T = 3.6 (1.0) | Multicomponent lifestyle intervention | 9 weeks | Passive control | Parent-reported questionnaire | Social Contextual Framework | Sleep duration, ST |
| 1. Hammersley ML et al. 2019 | Australia | RCT | ANZCTR 12616000119493 | T = 86; I = 42; C = 44 | I = 43.0%; C = 57.0% | I = 3.36 (0.80); C = 3.55 (1.02) | Multicomponent lifestyle intervention | 11 weeks | Passive control | ActiGraph GT3X+ | Social Cognitive Theory | Sleep duration, ST |
| 1. Hinkley T et al. 2015 | Australia | RCT | ACTRN12612000470897 | T = 22; I = 12; C = 10 | I = 33.0%; C = 40.0% | I = 2.94 (0.61); C = 2.85 (0.63) | ST reduction lifestyle intervention | 5 weeks | Wait-list control | activPAL | Social Cognitive Theory + Family Systems Theory | ST |
| 1. Hoffman JA et al. 2020 | United States | CRCT | NR | T = 57; I= 27; C = 30 | I = 51.9%; C = 70.0% | I = 4.75 (0.51); C = 4.62 (0.53) | PA – focused lifestyle intervention | 4 weeks | Usual care | ActiGraph GT9X | NR | MVPA |
| 1. Kaur N et al. 2024 | India | RCT | CTRI201709009761 | T = 340; I = 170; C = 170 | I = 49.4%; C = 48.8% | Range: 2–5 | ST reduction lifestyle intervention | 8 weeks | Usual care | Parent-reported questionnaire | Social Cognitive Theory + Self-Determination Theory + Socioecological Model (adapted) | ST |
| 1. Leis A et al. 2020 | Canada | CRCT | NCT02375490 | T = 339; I = 199; C = 140 | I = 51.0%; C = 54.0% | I = 4.1 (0.77); C = 4.1 (0.75) | Multicomponent lifestyle intervention | 8 months | Usual care | Actical accelerometer | Socioecological Model | TPA, LPA, MVPA, SB |
| 1. Lerner-Geva L et al. 2015 | Israel | CRCT | NR | T = 204; I A= 69; I B= 67; C = 68 | IA = 46.4%; IB = 55.2%; C = 45.6% | Range: 4–6 | IA: PA + nutrition lifestyle intervention  IB: Nutrition-focused lifestyle intervention | 4 months | Usual care | Parent-reported questionnaire | NR | Sleep duration, ST |
| 1. Lin YM et al. 2021 | China | CRCT | NCT04097587 | T = 129; I = 63; C = 66 | I = 58.7%; C = 47.0% | I = 5.6 (0.7); C = 5.6 (0.8) | ST reduction lifestyle intervention | 8 weeks | Wait-list control | Parent-reported questionnaire | Self-Efficacy Theory | ST |
| 1. Malden S et al. 2019 | United Kingdom | CRCT | ISRCTN12831555 | T = 42; I = 26; C = 16 | I = 38.5%; C = 43.8% | T = 4.4 (0.46) | Multicomponent lifestyle intervention | 18 weeks | Usual care | activPAL | NR | TPA, ST |
| 1. Marsh S et al. 2020 | New Zealand | RCT | ACTRN12618000823279 | T = 54; I = 27; C = 27 | I = 47.8%; C = 46.0% | I = 2.6 (0.7); C = 2.5 (0.7) | Multicomponent lifestyle intervention | 12 weeks | Wait-list control | Parent-reported questionnaire | Attachment Theory | Sleep duration, ST |
| 1. Martínez-Andrade GO et al. 2014 | Mexico | CRCT | NCT01539070 | T = 306; I = 168; C = 138 | I = 48.2%; C = 46.4% | I: 3.34 (0.84); C: 3.43 (0.83) | Multicomponent lifestyle intervention | 12 weeks | Usual care | Parent-reported questionnaire | NR | TPA, Sleep duration, ST |
| 1. Morgan PJ et al. 2022 | Australia | RCT | ACTRN12619000105145 | T = 125; I = 61; C = 64 | I = 44.3%; C = 34.4% | I = 4.0 (0.5); C = 3.9 (0.5) | Multicomponent lifestyle intervention | 10 weeks | Wait-list control | ActiGraph GT3X | NR | LPA, MVPA |
| 1. Nyström CD et al. 2017 | Sweden | RCT | NR | T = 313; I = 155; C = 158 | I = 45.0%; C = 47.0% | I = 4.5 (0.1); C = 4.5 (0.1) | Multicomponent lifestyle intervention | 6 months | Usual care | ActiGraph wGT3X-BT | Social Cognitive Theory + Behavior Change Techniques | SB, MVPA |
| 1. Østbye T et al. 2012 | United States | RCT | NCT00563264 | T = 400; I = 200; C = 200 | I = 43.5%; C = 45.0% | T = 3.06 (1.0) | Multicomponent lifestyle intervention | 8 months | Attention control | Actical accelerometer | Motivational Interviewing | SB, ST, MVPA |
| 1. Puder JJ et al. 2011 | Switzerland | CRCT | NCT00674544 | T = 652; I = 342; C = 310 | I = 49.0%; C = 51.0% | I = 5.2 (0.6); C = 5.2 (0.6) | Multicomponent lifestyle intervention | 10 months | Usual care | ActiGraph | NR | Sleep duration, ST |
| 1. Ray C et al. 2020 | Finland | CRCT | ISRCTN57165350 | T = 802; I = 442; C = 360 | I = 47.8%; C = 46.0% | I = 5.14 (1.04); C = 5.24 (1.06) | Multicomponent lifestyle intervention | 23 weeks | Usual care | ActiGraph wGT3X-BT | Self-Regulation Theory | TPA, ST |
| 1. Rifas-Shiman SL et al. 2017 | United States | CRCT | NR | T = 475; I = 271; C = 204 | I = 47.8%; C = 49.0% | I = 4.8 (1.2); C = 5.2 (1.1) | Multicomponent lifestyle intervention | 24 months | Usual care | Parent-reported questionnaire | Chronic Care Model | ST |
| 1. Sherwood NE et al. 2015 | United States | RCT | NCT01080885 | T = 60; I = 30; C = 30 | I = 50.0%; C = 40.0% | I = 2.60 (0.72); C = 2.90 (0.84) | Multicomponent lifestyle intervention | 6 months | Usual care | ActiGraph GT3X | Socioecological Model + Motivational Interviewing | TPA, MVPA |
| 1. Steenbock B et al. 2019 | Germany | CRCT | DRKS00011065 | T = 831; I = 440; C = 391 | I = 49.5%; C = 48.3% | I = 4.3 (0.8); C = 4.3 (0.8) | Multicomponent lifestyle intervention | 12 months | Usual care | Parent-reported questionnaire | NR | TPA, ST |
| 1. Taveras EM et al. 2011 | United States | CRCT | NR | T = 445; I = 253; C = 192 | I = 48.0%; C = 49.0% | I = 4.8 (1.2); C = 5.2 (1.1) | Multicomponent lifestyle intervention | 2 years | Usual care | Parent-reported questionnaire | Chronic Care Model | ST |
| 1. Tomayko EJ et al. 2018 | United States | RCT | NCT01776255 | T = 450; I = 225; C = 225 | I = 49.3%; C = 51.1% | I = 2.61 (0.76); C = 2.62 (0.65) | Multicomponent lifestyle intervention | 12 months | Usual care | Parent-reported questionnaire | NR | Sleep duration, ST |
| 1. van Grieken A et al. 2014 | Netherlands | CRCT | ISRCTN04965410 | T = 637; I = 349; C = 288 | I = 61.3%; C = 62.5% | I = 5.72 (0.42); C = 5.80 (0.45) | Multicomponent lifestyle intervention | 2 years | Usual care | Parent-reported questionnaire | Multi-Theory Behavior Change Framework | ST |
| 1. Walton K et al. 2016 | Canada | CRCT | NR | T = 54; I = 27; C = 27 | I = 55.6%; C = 57.1% | T = 3.0 (0.91) | Multicomponent lifestyle intervention | 9 weeks | Attention control | Parent-reported questionnaire | Social Contextual Framework | Sleep duration, ST |
| 1. Webster EK et al. 2023 | United States | CRCT | NR | T = 51; I = 32; C = 19 | I = 46.8%; C = 36.8% | I = 4.2 (0.6); C = 4.6 (0.7) | PA – focused lifestyle intervention | 8 weeks | Wait-list control | ActiGraph GT3X+ | NR | LPA, VPA, TPA, SB |
| 1. Wen X et al. 2018 | China | RCT | NR | T = 57; I = 29; C = 28 | I = 44.8%; C = 46.4% | I = 4.35 (0.28); C = 4.47 (0.31) | PA – focused lifestyle intervention | 10 weeks | Usual care | ActiGraph GT1M | NR | MVPA |
| 1. Yilmaz G et al. 2015 | Turkey | RCT | NR | T = 412; I = 211; C = 201 | I = 33.7%; C = 35.2% | I = 3.52 (1.28); C = 3.49 (1.22) | SB reduction lifestyle intervention | 9 months | Usual care | Parent-reported questionnaire | Social Cognitive Theory | ST |
| 1. Yoong SL et al. 2019 | Australia | RCT | ACTRN12616000822482 | T = 76; I = 38; C = 38 | I = 63.0%; C = 53.0% | I = 4.3 (0.5); C = 4.5 (0.6) | Sleep – focused lifestyle intervention | 3 months | Wait-list control | ActiGraph GT3X+ | Theory of Planned Behavior | TPA, MVPA, Sleep duration |

**Abbreviations: C, control; CRCT, cluster randomized controlled trial; I, intervention; IA/IB, intervention arm A/B; LPA, light physical activity; MPA, moderate physical activity; MVPA, moderate-to-vigorous physical activity; NR, not reported; PA, physical activity; RCT, randomized controlled trial; SB, sedentary behaviour; SD, standard deviation; ST, screen time; T, total; TPA, total physical activity; VPA, vigorous physical activity.**

1. Al-Walah MA, Donnelly M, Alhusaini AA, Heron N. Pre-school-based behaviour change intervention to increase physical activity levels amongst young children: a feasibility cluster randomised controlled trial. Front Public Health. 2024;12:1379582. Published 2024 May 2. doi:10.3389/fpubh.2024.1379582
2. Barber SE, Jackson C, Hewitt C, et al. Assessing the feasibility of evaluating and delivering a physical activity intervention for pre-school children: a pilot randomised controlled trial. Pilot Feasibility Stud. 2016;2:12. Published 2016 Feb 18. doi:10.1186/s40814-016-0052-4
3. Bonis M, Loftin M, Ward D, Tseng TS, Clesi A, Sothern M. Improving physical activity in daycare interventions. Child Obes. 2014;10(4):334-341. doi:10.1089/chi.2014.0040
4. Byrd-Bredbenner C, Martin-Biggers J, Povis GA, Worobey J, Hongu N, Quick V. Promoting healthy home environments and lifestyles in families with preschool children: HomeStyles, a randomized controlled trial. Contemp Clin Trials. 2018;64:139-151. doi:10.1016/j.cct.2017.10.012
5. Chow AF, Leis A, Humbert L, Muhajarine N, Engler-Stringer R. Healthy Start-Départ Santé: A pilot study of a multilevel intervention to increase physical activity, fundamental movement skills and healthy eating in rural childcare centres. Can J Public Health. 2016;107(3):e312-e318. doi:10.17269/CJPH.107.5279
6. De Bock F, Genser B, Raat H, Fischer JE, Renz-Polster H. A participatory physical activity intervention in preschools: a cluster randomized controlled trial. Am J Prev Med. 2013;45(1):64-74. doi:10.1016/j.amepre.2013.01.032
7. De Coen V, De Bourdeaudhuij I, Vereecken C, et al. Effects of a 2-year healthy eating and physical activity intervention for 3-6-year-olds in communities of high and low socio-economic status: the POP (Prevention of Overweight among Pre-school and school children) project. Public Health Nutr. 2012;15(9):1737-1745. doi:10.1017/S1368980012000687
8. Downing KL, Salmon J, Hinkley T, Hnatiuk JA, Hesketh KD. Feasibility and Efficacy of a Parent-Focused, Text Message-Delivered Intervention to Reduce Sedentary Behavior in 2- to 4-Year-Old Children (Mini Movers): Pilot Randomized Controlled Trial. JMIR Mhealth Uhealth. 2018;6(2):e39. Published 2018 Feb 9. doi:10.2196/mhealth.8573
9. Feng J, Huang WY, Sit CH, Reilly JJ, Khan A. Effectiveness of a parent-focused intervention targeting 24-hour movement behaviours in preschool-aged children: a randomised controlled trial. Int J Behav Nutr Phys Act. 2024;21(1):98. Published 2024 Sep 9. doi:10.1186/s12966-024-01650-2
10. Fitzgibbon ML, Stolley MR, Schiffer L, Van Horn L, KauferChristoffel K, Dyer A. Two-year follow-up results for Hip-Hop to Health Jr.: a randomized controlled trial for overweight prevention in preschool minority children. J Pediatr. 2005;146(5):618-625. doi:10.1016/j.jpeds.2004.12.019
11. Fitzgibbon ML, Stolley MR, Schiffer L, Van Horn L, KauferChristoffel K, Dyer A. Hip-Hop to Health Jr. for Latino preschool children. Obesity (Silver Spring). 2006;14(9):1616-1625. doi:10.1038/oby.2006.186
12. Fitzgibbon ML, Stolley MR, Schiffer LA, et al. Hip-Hop to Health Jr. Obesity Prevention Effectiveness Trial: postintervention results. Obesity (Silver Spring). 2011;19(5):994-1003. doi:10.1038/oby.2010.314
13. Fitzgibbon ML, Stolley MR, Schiffer L, et al. Family-based hip-hop to health: outcome results. Obesity (Silver Spring). 2013;21(2):274-283. doi:10.1002/oby.20269
14. French SA, Sherwood NE, Veblen-Mortenson S, et al. Multicomponent Obesity Prevention Intervention in Low-Income Preschoolers: Primary and Subgroup Analyses of the NET-Works Randomized Clinical Trial, 2012-2017. Am J Public Health. 2018;108(12):1695-1706. doi:10.2105/AJPH.2018.304696
15. Goldfield GS, Harvey ALJ, Grattan KP, et al. Effects of Child Care Intervention on Physical Activity and Body Composition. Am J Prev Med. 2016;51(2):225-231. doi:10.1016/j.amepre.2016.03.024
16. Haines J, McDonald J, O'Brien A, et al. Healthy Habits, Happy Homes: randomized trial to improve household routines for obesity prevention among preschool-aged children. JAMA Pediatr. 2013;167(11):1072-1079. doi:10.1001/jamapediatrics.2013.2356
17. Haines J, Rifas-Shiman SL, Gross D, McDonald J, Kleinman K, Gillman MW. Randomized trial of a prevention intervention that embeds weight-related messages within a general parenting program. Obesity (Silver Spring). 2016;24(1):191-199. doi:10.1002/oby.21314
18. Hammersley ML, Okely AD, Batterham MJ, Jones RA. An Internet-Based Childhood Obesity Prevention Program (Time2bHealthy) for Parents of Preschool-Aged Children: Randomized Controlled Trial. J Med Internet Res. 2019;21(2):e11964. Published 2019 Feb 8. doi:10.2196/11964
19. Hinkley T, Cliff DP, Okely AD. Reducing electronic media use in 2-3 year-old children: feasibility and efficacy of the Family@play pilot randomised controlled trial. BMC Public Health. 2015;15:779. Published 2015 Aug 14. doi:10.1186/s12889-015-2126-2
20. Hoffman JA, Schmidt EM, Arguello DJ, et al. Online preschool teacher training to promote physical activity in young children: A pilot cluster randomized controlled trial. Sch Psychol. 2020;35(2):118-127. doi:10.1037/spq0000349
21. Kaur N, Gupta M, Chakrapani V, et al. Effectiveness of a program to lower unwanted media screens among 2-5-year-old children: a randomized controlled trial. Front Public Health. 2024;12:1304861. Published 2024 Jun 18. doi:10.3389/fpubh.2024.1304861
22. Leis A, Ward S, Vatanparast H, et al. Effectiveness of the Healthy Start-Départ Santé approach on physical activity, healthy eating and fundamental movement skills of preschoolers attending childcare centres: a randomized controlled trial. BMC Public Health. 2020;20(1):523. Published 2020 Apr 19. doi:10.1186/s12889-020-08621-9
23. Lerner-Geva L, Bar-Zvi E, Levitan G, Boyko V, Reichman B, Pinhas-Hamiel O. An intervention for improving the lifestyle habits of kindergarten children in Israel: a cluster-randomised controlled trial investigation. Public Health Nutr. 2015;18(9):1537-1544. doi:10.1017/S136898001400024X
24. Lin YM, Kuo SY, Chang YK, et al. Effects of Parental Education on Screen Time, Sleep Disturbances, and Psychosocial Adaptation Among Asian Preschoolers: A Randomized Controlled Study. J Pediatr Nurs. 2021;56:e27-e34. doi:10.1016/j.pedn.2020.07.003
25. Malden S, Reilly JJ, Gibson AM, et al. A feasibility cluster randomised controlled trial of a preschool obesity prevention intervention: ToyBox-Scotland. Pilot Feasibility Stud. 2019;5:128. Published 2019 Nov 9. doi:10.1186/s40814-019-0521-7
26. Marsh S, Taylor R, Galland B, Gerritsen S, Parag V, Maddison R. Results of the 3 Pillars Study (3PS), a relationship-based programme targeting parent-child interactions, healthy lifestyle behaviours, and the home environment in parents of preschool-aged children: A pilot randomised controlled trial. PLoS One. 2020;15(9):e0238977. Published 2020 Sep 17. doi:10.1371/journal.pone.0238977
27. Martínez-Andrade GO, Cespedes EM, Rifas-Shiman SL, et al. Feasibility and impact of Creciendo Sanos, a clinic-based pilot intervention to prevent obesity among preschool children in Mexico City. BMC Pediatr. 2014;14:77. Published 2014 Mar 20. doi:10.1186/1471-2431-14-77
28. Morgan PJ, Grounds JA, Ashton LM, et al. Impact of the 'Healthy Youngsters, Healthy Dads' program on physical activity and other health behaviours: a randomised controlled trial involving fathers and their preschool-aged children. BMC Public Health. 2022;22(1):1166. Published 2022 Jun 10. doi:10.1186/s12889-022-13424-1
29. Nyström CD, Sandin S, Henriksson P, et al. Mobile-based intervention intended to stop obesity in preschool-aged children: the MINISTOP randomized controlled trial. Am J Clin Nutr. 2017;105(6):1327-1335. doi:10.3945/ajcn.116.150995
30. Østbye T, Krause KM, Stroo M, et al. Parent-focused change to prevent obesity in preschoolers: results from the KAN-DO study. Prev Med. 2012;55(3):188-195. doi:10.1016/j.ypmed.2012.06.005
31. Puder JJ, Marques-Vidal P, Schindler C, et al. Effect of multidimensional lifestyle intervention on fitness and adiposity in predominantly migrant preschool children (Ballabeina): cluster randomised controlled trial. BMJ. 2011;343:d6195. Published 2011 Oct 13. doi:10.1136/bmj.d6195
32. Ray C, Figuereido R, Vepsäläinen H, et al. Effects of the Preschool-Based Family-Involving DAGIS Intervention Program on Children's Energy Balance-Related Behaviors and Self-Regulation Skills: A Clustered Randomized Controlled Trial. Nutrients. 2020;12(9):2599. Published 2020 Aug 26. doi:10.3390/nu12092599
33. Rifas-Shiman SL, Taveras EM, Gortmaker SL, et al. Two-year follow-up of a primary care-based intervention to prevent and manage childhood obesity: the High Five for Kids study. Pediatr Obes. 2017;12(3):e24-e27. doi:10.1111/ijpo.12141
34. Sherwood NE, JaKa MM, Crain AL, Martinson BC, Hayes MG, Anderson JD. Pediatric Primary Care-Based Obesity Prevention for Parents of Preschool Children: A Pilot Study. Child Obes. 2015;11(6):674-682. doi:10.1089/chi.2015.0009
35. Steenbock B, Buck C, Zeeb H, Rach S, Pischke CR. Impact of the intervention program "JolinchenKids - fit and healthy in daycare" on energy balance related-behaviors: results of a cluster controlled trial. BMC Pediatr. 2019;19(1):432. Published 2019 Nov 13. doi:10.1186/s12887-019-1817-8
36. Taveras EM, Gortmaker SL, Hohman KH, et al. Randomized controlled trial to improve primary care to prevent and manage childhood obesity: the High Five for Kids study. Arch Pediatr Adolesc Med. 2011;165(8):714-722. doi:10.1001/archpediatrics.2011.44
37. Tomayko EJ, Prince RJ, Cronin KA, Kim K, Parker T, Adams AK. The Healthy Children, Strong Families 2 (HCSF2) Randomized Controlled Trial Improved Healthy Behaviors in American Indian Families with Young Children. Curr Dev Nutr. 2018;3(Suppl 2):53-62. Published 2018 Nov 16. doi:10.1093/cdn/nzy087
38. van Grieken A, Renders CM, Veldhuis L, Looman CW, Hirasing RA, Raat H. Promotion of a healthy lifestyle among 5-year-old overweight children: health behavior outcomes of the 'Be active, eat right' study. BMC Public Health. 2014;14:59. Published 2014 Jan 21. doi:10.1186/1471-2458-14-59
39. Walton K, Filion AJ, Gross D, et al. Parents and Tots Together: Pilot randomized controlled trial of a family-based obesity prevention intervention in Canada. Can J Public Health. 2016;106(8):e555-e562. Published 2016 Mar 16. doi:10.17269/cjph.106.5224
40. Webster EK, Kepper MM, Saha S, et al. Painted playgrounds for preschoolers' physical activity and fundamental motor skill improvement: a randomized controlled pilot trial of effectiveness. BMC Pediatr. 2023;23(1):455. Published 2023 Sep 9. doi:10.1186/s12887-023-04260-2
41. Wen X, Zhang Y, Gao Z, Zhao W, Jie J, Bao L. Effect of Mini-Trampoline Physical Activity on Executive Functions in Preschool Children. Biomed Res Int. 2018;2018:2712803. Published 2018 May 10. doi:10.1155/2018/2712803
42. Yilmaz G, Demirli Caylan N, Karacan CD. An intervention to preschool children for reducing screen time: a randomized controlled trial. Child Care Health Dev. 2015;41(3):443-449. doi:10.1111/cch.12133
43. Yoong SL, Grady A, Stacey F, et al. A pilot randomized controlled trial examining the impact of a sleep intervention targeting home routines on young children's (3-6 years) physical activity. Pediatr Obes. 2019;14(4):e12481. doi:10.1111/ijpo.12481
