# Supplementary material for: Lifestyle interventions and 24-hour movement behaviors in preschool children: a systematic review and meta-analysis
Source: Front Public Health. 2026 Jun 17;14:1846736. doi: 10.3389/fpubh.2026.1846736 (PMC13318789; doi:10.3389/fpubh.2026.1846736)
Supplement: Supplementary file 17 [file Table_6.docx]

**Summary of finding table from the GRADE profiler**

**Question: Effect of lifestyle interventions on 24-hour behaviors in preschool children**

| **Certainty assessment** | |  |  |  |  | **№ of patients** | | **Effect** | | **Certainty** |
| --- | --- | --- | --- | --- | --- | --- | --- | --- | --- | --- |
| **№ of studies** | **Risk of bias** | **Inconsistency** | **Indirectness** | **Imprecision** | **Other considerations** | **intervention** | **comparison** | **Relative (95% CI)** | **Absolute (95% CI)** |  |
| **Total physical activity (TPA)** | | | | | | | | | | |
| Thirteen | serious | serious | not serious | not serious | none | 1420 | 1326 | - | 7.83 min/day higher  (−0.42, 16.09) | ⨁⨁◯◯  Low ^a,b^ |
| **Vigorous physical activity (VPA)** | | | | | | | | | | |
| Three | serious | serious | not serious | not serious | none | 401 | 381 | - | 0.34 min/day higher  (−2.40, 3.07) | ⨁⨁◯◯  Low ^a,b^ |
| **Moderate-to-vigorous physical activity (MVPA)** | | | | | | | | | | |
| Three | serious | serious | not serious | not serious | none | 1920 | 1770 | - | 5.74 min/day higher  (2.27, 9.21) | ⨁⨁◯◯  Low ^a,b^ |
| **Moderate physical activity (MPA)** | | | | | | | | | | |
| Twenty | serious | serious | not serious | not serious | none | 419 | 398 | - | 1.24 min/day higher  (-3.09, 5.57) | ⨁⨁◯◯  Low ^a,b^ |
| **Light physical activity (LPA)** | | | | | | | | | | |
| Eight | serious | serious | not serious | not serious | none | 748 | 680 | - | 0.71 min/day higher  (−2.70, 4.12) | ⨁⨁◯◯  Low ^a,b^ |
| **Screen time (ST)** | | | | | | | | | | |
| Thirty-five | serious | serious | not serious | not serious | none | 5503 | 4819 | - | -0.33 h/day lower  (-0.53, -0.13) | ⨁⨁◯◯  Low ^a,b^ |
| **Sedentary behaviour (SB)** | | | | | | | | | | |
| Thirteen | serious | serious | not serious | not serious | none | 1601 | 1459 | - | -8.18 min/day lower  (-15.77, -0.59) | ⨁⨁◯◯  Low ^a,b^ |
| **Sleep duration** | | | | | | | | | | |
| Fourteen | serious | serious | not serious | not serious | none | 1265 | 1222 | - | 0.18 h/day higher  (0.01, 0.35) | ⨁⨁◯◯  Low ^a,b^ |

Abbreviations: GRADE, Grading of Recommendations, Assessment, Development and Evaluation; CI, confidence interval.

Explanation:

^a^ Risk of bias: downgraded one level because the majority of included trials were judged as “some concerns” and a meaningful proportion were at high risk of bias

^b^ Inconsistency: downgraded one level due to substantial heterogeneity (high I²) across studies, reflecting variability in effect estimates.
